# Mitochondrial Ca^2+^-coupled generation of reactive oxygen species, peroxynitrite formation, and endothelial dysfunction in Cantú syndrome

**DOI:** 10.1172/jci.insight.176212

**Published:** 2024-08-01

**Authors:** Elsayed Metwally, Alfredo Sanchez Solano, Boris Lavanderos, Evan Yamasaki, Pratish Thakore, Conor McClenaghan, Natalia Rios, Rafael Radi, Yumei Feng Earley, Colin G. Nichols, Scott Earley

**Affiliations:** 1 Department of Pharmacology, Center for Molecular and Cellular Signaling in the Cardiovascular System, University of Nevada, Reno School of Medicine, Reno, Nevada, USA.; 2 Department of Cytology and Histology, Faculty of Veterinary Medicine, Suez Canal University, Ismailia, Egypt.; 3 Departments of Pharmacology and Medicine, Center for Advanced Biotechnology and Medicine, Robert Wood Johnson Medical School, Rutgers University, Piscataway, New Jersey, USA.; 4 Departamento de Bioquímica, Facultad de Medicina, and; 5 Centro de Investigaciones Biomédicas (CEINBIO), Facultad de Medicina, Universidad de la República, Montevideo, Uruguay.; 6 Center for the Investigation of Membrane Excitability Diseases and Departments of Cell Biology and Physiology, Washington University School of Medicine, St. Louis, Missouri, USA.

**Keywords:** Cell biology, Vascular biology, Calcium signaling, Cardiovascular disease, Ion channels

## Abstract

Cantú syndrome is a multisystem disorder caused by gain-of-function (GOF) mutations in *KCNJ8* and *ABCC9,* the genes encoding the pore-forming inward rectifier Kir6.1 and regulatory sulfonylurea receptor SUR2B subunits, respectively, of vascular ATP-sensitive K^+^ (K_ATP_) channels. In this study, we investigated changes in the vascular endothelium in mice in which Cantú syndrome–associated *Kcnj8* or *Abcc9* mutations were knocked in to the endogenous loci. We found that endothelium-dependent dilation was impaired in small mesenteric arteries from Cantú mice. Loss of endothelium-dependent vasodilation led to increased vasoconstriction in response to intraluminal pressure or treatment with the adrenergic receptor agonist phenylephrine. We also found that either K_ATP_ GOF or acute activation of K_ATP_ channels with pinacidil increased the amplitude and frequency of wave-like Ca^2+^ events generated in the endothelium in response to the vasodilator agonist carbachol. Increased cytosolic Ca^2+^ signaling activity in arterial endothelial cells from Cantú mice was associated with elevated mitochondrial [Ca^2+^] and enhanced reactive oxygen species (ROS) and peroxynitrite levels. Scavenging intracellular or mitochondrial ROS restored endothelium-dependent vasodilation in the arteries of mice with K_ATP_ GOF mutations. We conclude that mitochondrial Ca^2+^ overload and ROS generation, which subsequently leads to nitric oxide consumption and peroxynitrite formation, cause endothelial dysfunction in mice with Cantú syndrome.

## Abstract 2

Metwally et al. show that mitochondrial Ca^2+^ overload and ROS generation, which subsequently leads to nitric oxide consumption and peroxynitrite formation, cause endothelial dysfunction in mice with Cantú syndrome

## Graphical abstract

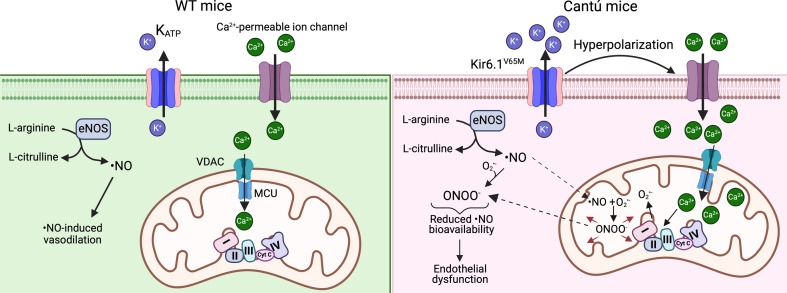

## Introduction

Cantú syndrome is a rare genetic disorder characterized by hypertrichosis, distinctive facial features, osteochondrodysplasia, edema, and cardiovascular abnormalities (1–5). The disease is caused by gain-of-function (GOF) mutations in *KCNJ8* or *ABCC9* genes encoding the pore-forming (Kir6.1) and regulatory (SUR2B) subunits, respectively, of ATP-sensitive K^+^ (K_ATP_) channels (6–9), with severity varying among patients with Cantú, likely due to the differential effects of various mutations on channel function (10). Cantú mutations either decrease K_ATP_ channel relative sensitivity to inhibitory adenosine 5′-triphosphate (ATP) or increase sensitivity to activating adenosine 5′-diphosphate (ADP) (11). Interestingly, the SUR2 [S1054Y] mutation in the ankyrin B domain of *ABCC9* was shown to augment K_ATP_ activation while reducing membrane expression of the channel, which might moderate the clinical phenotype (12). Mice expressing *Kcnj8* or *Abcc9* GOF mutations corresponding to those found in some patients with Cantú replicate the primary cardiovascular aberrations of the disease, including aortic dilation, cardiac hypertrophy, and increased carotid artery compliance (2). Kir6.1 and SUR2B K_ATP_ channel subunits are present in vascular endothelial cells (13–15), and studies using endothelial-specific Kir6.1-KO mice have shown that endothelial K_ATP_ channels contribute to vascular reactivity and blood pressure control (14, 16). However, the direct effects of chronically elevated K_ATP_ activity on endothelial cell function have not been previously described. Accordingly, we here used CRISPR/Cas9–generated *Kcnj8* and *Abcc9* mutant mice to investigate the effects of K_ATP_ GOF on the vascular endothelium.

Endothelial cells influence the membrane potential and contractility of overlying smooth muscle cells (SMCs) by releasing vasoactive substances, such as nitric oxide (·NO), and by direct electrical communication through myoendothelial gap junctions (17, 18). Both processes are regulated by local and global changes in endothelial cell cytosolic Ca^2+^ (19). Endothelium-dependent vasodilator agonists, such as acetylcholine and carbachol (CCh), engage vasodilator pathways by initiating Ca^2+^ influx and Ca^2+^ release from the endoplasmic reticulum (ER) (18, 20). K^+^ channel activity influences this process by hyperpolarizing the endothelial cell plasma membrane and increasing the electrochemical gradient for Ca^2+^ influx (21, 22). Therefore, we predicted that Cantú mutations would enhance agonist-induced Ca^2+^ influx and, consequently, augment endothelium-dependent vasodilation.

Contrary to these predictions, we found evidence for endothelial dysfunction in mice with K_ATP_ GOF mutations. Although small mesenteric arteries from Cantú mice displayed exaggerated agonist-induced endothelial cell Ca^2+^-signaling activity, endothelium-dependent vasodilation was essentially abolished in these arteries. Loss of the tonic vasodilator influence from the endothelium caused isolated arteries from K_ATP_ GOF mice to become hypercontractile. Additional mechanistic studies suggested that excessive cytosolic Ca^2+^ signaling increases mitochondrial Ca^2+^ levels, leading to an elevation in reactive oxygen species (ROS) that, in turn, diminishes ·NO bioavailability and triggers peroxynitrite (OONO^–^) formation that impairs endothelium-dependent vasodilation. Thus, endothelial dysfunction emerges as a consequence of Cantú syndrome.

## Results

### Elevated K_ATP_ channel activity in endothelial cells from Cantú mice hyperpolarizes the resting membrane potential.

**Figure 1:**
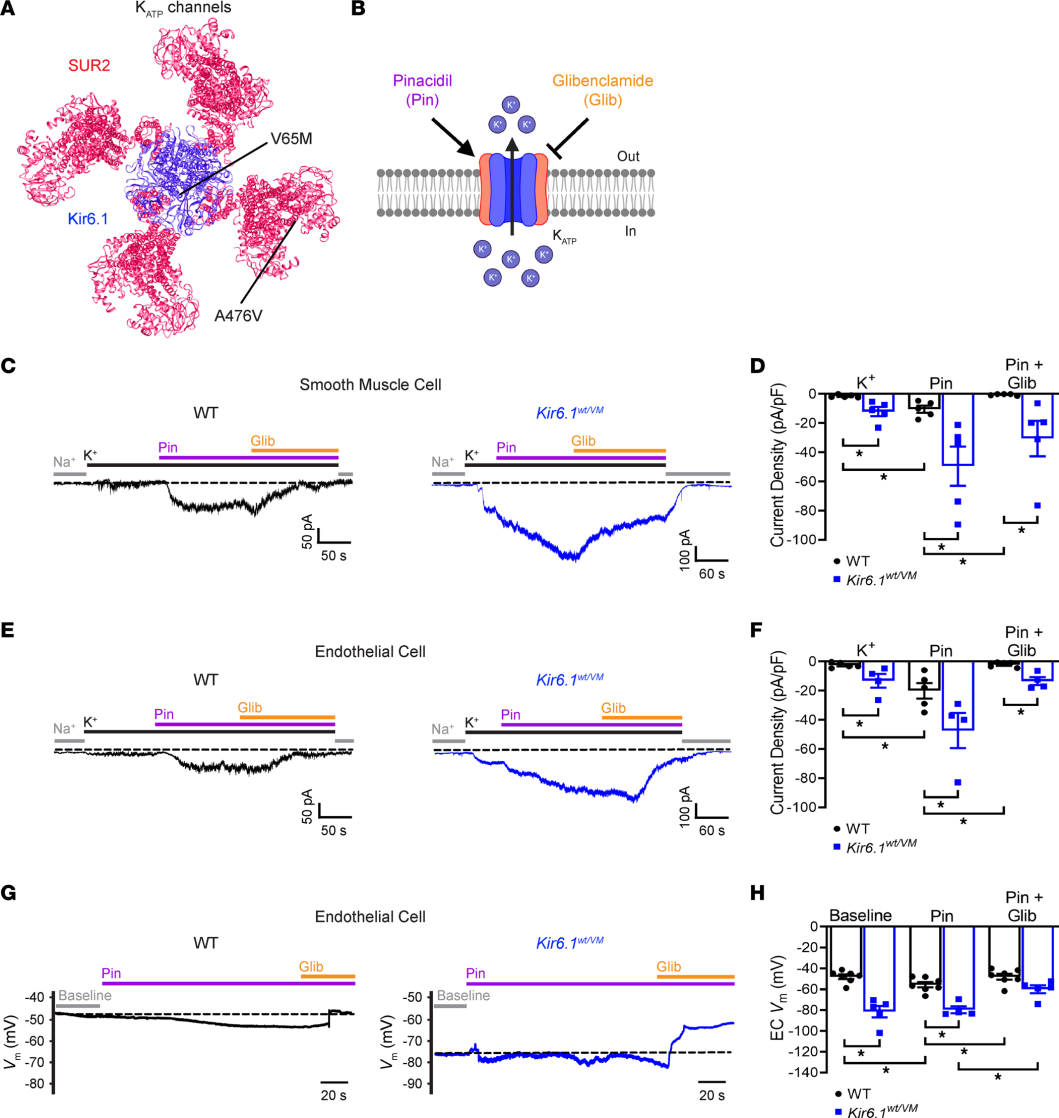
Elevated K_ATP_ channel activity in endothelial cells from Cantú mice hyperpolarizes the resting membrane potential. (**A**) Structural representation of the Kir6.2/SUR1 K_ATP_ channel (PDB ID: 5WUA) with homologous positions of Kir6.1 [V65M] and SUR2 [A476V] mutations in mice indicated. (**B**) Schematic diagram illustrating K_ATP_ channel activation by the synthetic opener pinacidil (Pin) and inhibition by the sulfonylurea compound glibenclamide (Glib). (**C**) Representative whole-cell voltage-clamp recordings from acutely isolated mesenteric vascular SMCs from WT and Kir6.1^wt/VM^ mice. (**D**) Summary data of whole-cell current densities from voltage-clamp recordings of WT and Kir6.1^wt/VM^ SMCs showing significant increases in basal and pinacidil-activated K_ATP_ conductance, which are resistant to glibenclamide in Kir6.1^wt/VM^ Data are presented as means ± SEM (*n* = 5 cells from 3 animals per group; *P < 0.05, 2-way ANOVA with Tukey’s post hoc test). (**E**) Representative whole-cell voltage-clamp recordings of freshly isolated mesenteric vascular endothelial cells from WT and Kir6.1^wt/VM^ mice. (**F**) Summary data of whole-cell current densities from voltage-clamp recordings of vascular endothelial cells from WT and Kir6.1^wt/VM^ with indicated treatments. Data are presented as means ± SEM (*n* = 4–5 cells from 3 animals per group; *P < 0.05, 2-way ANOVA with Tukey’s post hoc test). (**G**) Representative whole-cell current-clamp recordings from freshly isolated mesenteric vascular endothelial cells isolated from WT and Kir6.1^wt/VM^ mice. Recordings were recorded initially under basal conditions and then following treatment with pinacidil and glibenclamide. (**H**) Summary of current-clamp recordings. Data are presented as means ± SEM (*n* = 5–7 cells from 3 animals per group; *P < 0.05, 2-way ANOVA with Tukey’s post hoc test).

Mice heterozygous for the CRISPR/Cas9–generated (V65M) GOF mutation in *Kcnj8* (Kir6.1^wt/VM^) or homozygous for the (A476V) GOF mutation in *Abcc9* (SUR^AV/AV^) were used throughout this study (Figure 1A) (2). Heterozygous Kir6.1^wt/VM^ mice were used because homozygous Kir6.1^VM/VM^ mice die by around day 20. The majority of homozygous SUR^AV/AV^ mice survive to adulthood. WT littermates were used as controls for both strains. The effect of the Kir6.1 mutation on K_ATP_ channel function was first determined using whole-cell patch-clamp electrophysiology on freshly isolated mesenteric artery SMCs and endothelial cells. Currents were initially measured at a holding potential of 70 mV in a high-Na^+^ bath solution before switching to a high-K^+^ bath solution containing the K_ATP_ channel activator pinacidil and then a bath solution containing pinacidil and the K_ATP_ channel inhibitor glibenclamide (Figure 1B). The recordings revealed that basal K^+^ currents were greater in SMCs from Kir6.1^wt/VM^ mice than in WT controls (Figure 1, C and D). Application of the K_ATP_ channel opener pinacidil increased K^+^ currents in SMCs from both groups, but this effect was greater in SMCs from Kir6.1^wt/VM^ mice, which was in agreement with prior studies (2, 23). Subsequent application of the K_ATP_ channel inhibitor glibenclamide essentially abolished pinacidil-activated currents in SMCs from WT animals, but, consistent with prior reports (2, 23), was less effective in cells from Kir6.1^wt/VM^ mice (Figure 1, C and D).

Mesenteric artery endothelial cells from *Vec*^cre^ x *mT/mG* reporter mice (24) expressing eGFP or selectively labeled with isolectin-IB4 were enzymatically isolated for whole-cell patch-clamp electrophysiology experiments. K_ATP_ channel activity was greater in endothelial cells from Kir6.1^wt/VM^ mice compared with controls (Figure 1, E and F). Pinacidil increased the current in both groups, an effect that was again greater in endothelial cells from Kir6.1^wt/VM^ mice. Glibenclamide nearly abolished pinacidil-activated currents in endothelial cells from control mice but was less effective in cells from Kir6.1^wt/VM^ mice (Figure 1, E and F). These data demonstrate that K_ATP_ channel activity is elevated in native endothelial cells from Kir6.1^wt/VM^ mice.

Using current-clamp electrophysiology to measure the resting membrane potential (*V_m_*) of isolated native mesenteric endothelial cells, we found that the *V_m_* of endothelial cells from Kir6.1^wt/VM^ mice (–81.5 ± 5.5 mV) was significantly hyperpolarized compared with that in cells from controls (–48.0 ± 2.1 mV) (Figure 1, G and H). Pinacidil hyperpolarized the plasma membrane of cells from WT animals, and glibenclamide reversed this effect. Interestingly, pinacidil did not further hyperpolarize the membrane of endothelial cells from Kir6.1^wt/VM^ mice (Figure 1, G and H). However, glibenclamide depolarized the membrane potential of cells from mutant mice (Figure 1, G and H), indicating that elevated K_ATP_ channel activity in endothelial cells from Kir6.1^wt/VM^ mice maintains a hyperpolarized resting membrane potential.

### Cantú mice exhibit endothelial dysfunction.

**Figure 2:**
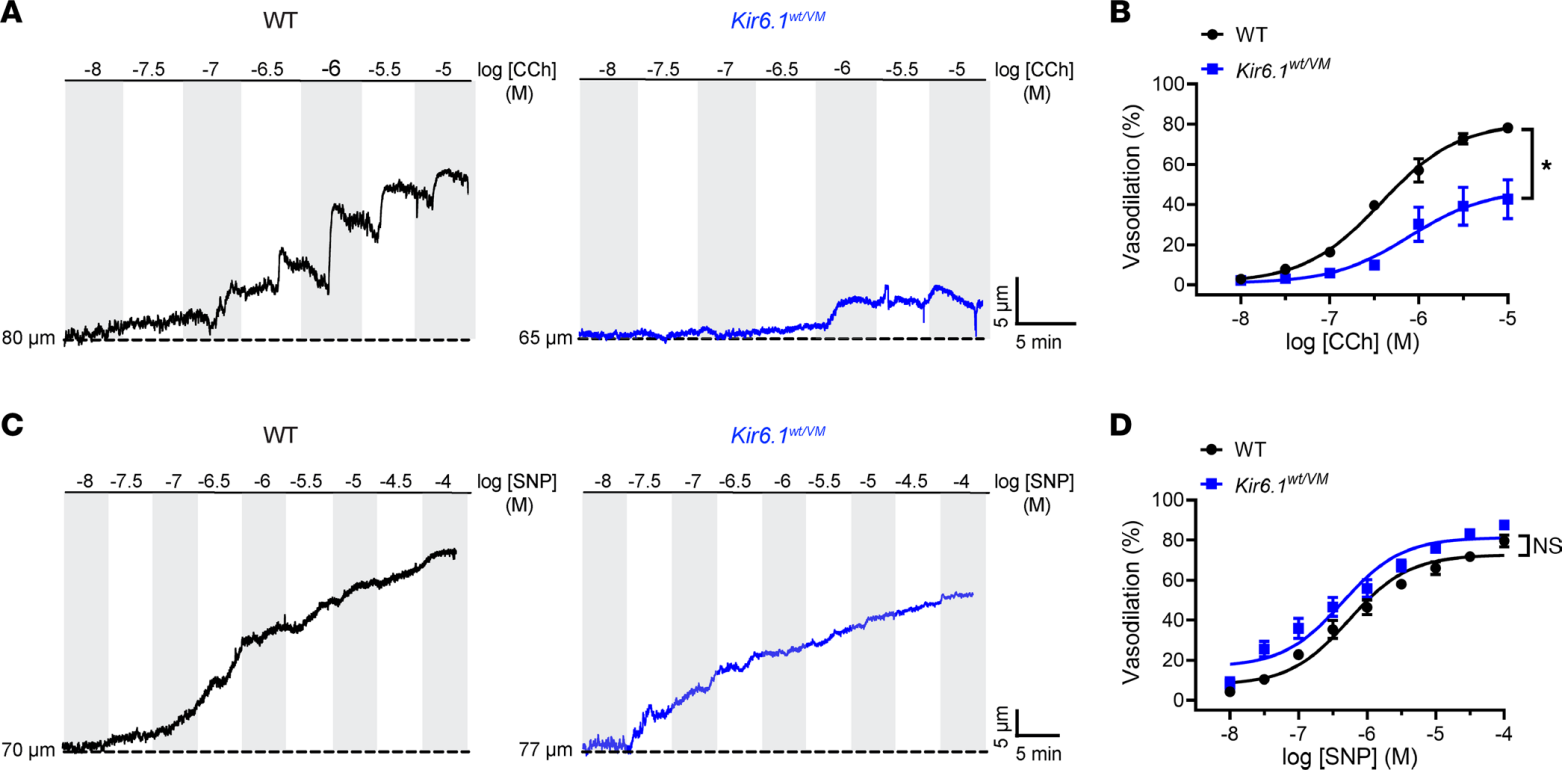
Cantú mice exhibit endothelial dysfunction. (**A**) Representative recordings and (**B**) summary data showing endothelium-dependent vasodilation evoked by the muscarinic receptor agonist carbachol (CCh) in mesenteric arterioles from WT and Kir6.1^wt/VM^ mice. Data are presented as means ± SEM (*n* = 3–5 vessels from 3 animals per group; *P < 0.05, 2-way ANOVA with Šídák’s multiple comparisons test). (**C**) Representative recordings and (**D**) summary data showing vasodilatory responses to the NO donor SNP in isolated mesenteric arteries from WT and Kir6.1^wt/VM^ mice. Data are presented as means ± SEM (*n* = 3–5 vessels from 3 animals per group; 2-way ANOVA with Šídák’s multiple comparisons test).

The endothelial function of mesenteric arteries from Cantú mutant mice was evaluated using pressure myography. Third-order mesenteric arteries were pressurized to 20 mmHg and preconstricted by applying the thromboxane A_2_ receptor agonist U46619. Arteries were then exposed to increasing concentrations of CCh, a muscarinic receptor agonist and endothelium-dependent vasodilator. CCh-induced vasodilation was significantly blunted in arteries from Kir6.1^wt/VM^ mice compared with controls (Figure 2, A and B), indicating that the endothelium was dysfunctional. In control studies, we found that vasodilation in response to the ·NO donor sodium nitroprusside (SNP) did not differ between Kir6.1^wt/VM^ and WT mice (Figure 2, C and D), indicating that SMCs in arteries from mutant mice retain the ability to relax in response to ·NO. Similarly, endothelium-dependent vasodilation in response to CCh was significantly blunted in arteries from SUR^AV/AV^ mice, but vasodilation in response to SNP remained intact (Supplemental Figure 1; supplemental material available online with this article; https://doi.org/10.1172/jci.insight.176212DS1). These data demonstrate endothelial dysfunction in mesenteric arteries from both *Kcnj8* and *Abcc9* Cantú K_ATP_ GOF mice.

### Small mesenteric arteries from Cantú mice are hypercontractile.

Endothelial dysfunction, a hallmark of multiple cardiovascular diseases, is characterized by elevated vascular tone reflecting withdrawal of the tonic relaxing influence of the endothelium (25, 26). To determine the effects of Cantú-associated endothelial dysfunction on vascular contractility, we assessed spontaneous myogenic tone generated by mesenteric arteries using pressure myography (27). The active contraction of SMCs was determined by measuring changes in diameter following stepwise increases in intraluminal pressure from 5 to 100 mmHg. The pressure was then lowered back to 5 mmHg and vessels were superfused with a Ca^2+^-free solution, after which passive changes in diameter in the absence of muscular contraction were recorded at each pressure. Myogenic tone was calculated as the difference between active and passive diameters, normalized to the passive diameter (27).

**Figure 3:**
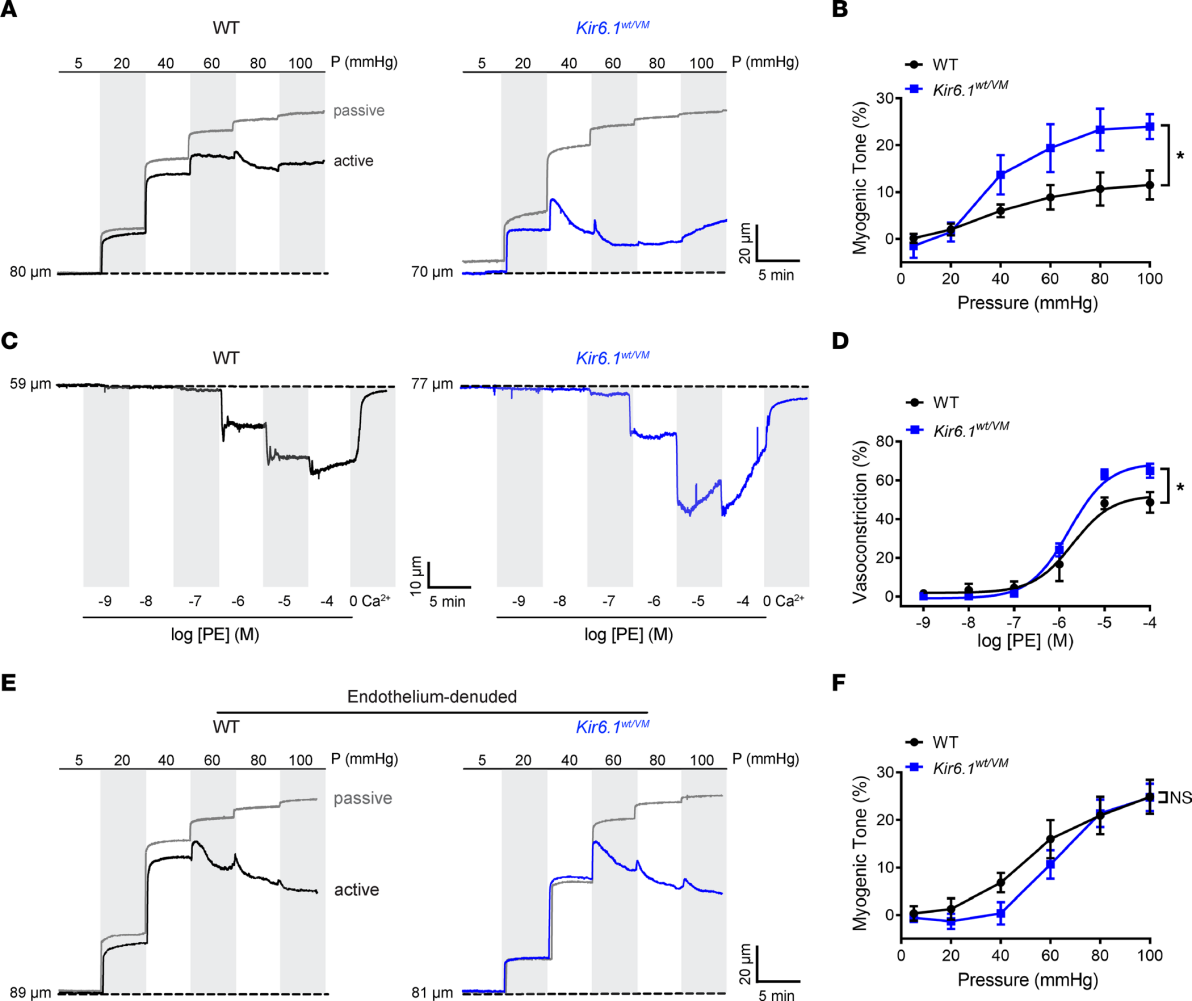
Small mesenteric arteries from Cantú mice are hypercontractile. (**A**) Representative recordings of the luminal diameter of isolated mesenteric arteries from WT and Kir6.1^wt/VM^ mice showing changes in lumen diameter in response to increases in intraluminal pressure under active and passive (Ca^2+^-free) conditions. (**B**) Summary data of myogenic tone from WT and Kir6.1^wt/VM^ mice. Data are presented as means ± SEM (*n* = 6–7 vessels from 4 animals per group; *P < 0.05, 2-way ANOVA with Šídák’s multiple comparisons test). (**C**) Representative recordings and (**D**) summary data showing constriction of mesenteric arteries from WT and Kir6.1^wt/VM^ mice in response to increasing concentrations of the α1-adrenoceptor agonist phenylephrine (PE). Data are presented as means ± SEM (*n* = 4–5 for Kir6.1^wt/VM^ from 3 animals per group; *P < 0.05, 2-way ANOVA with Šídák’s multiple comparisons test). (**E)** Representative recording and (**F**) summary data of myogenic tone showing changes in the lumen diameter of endothelium-denuded mesenteric arteries from WT and Kir6.1^wt/VM^ mice. Data are presented as means ± SEM (*n* = 7–9 vessels from 6 animals per group; 2-way ANOVA with Šídák’s multiple comparisons test).

Mesenteric arteries isolated from Kir6.1^wt/VM^ mice exhibited greater constriction in response to all pressures greater than 20 mmHg, and myogenic tone was significantly higher in these arteries than in those from control mice (Figure 3, A and B). Constriction in response to the α1-adrenoceptor agonist phenylephrine (PE) was also significantly greater in arteries from Kir6.1^wt/VM^ mice compared with WT controls (Figure 3, C and D). Similarly, arteries from SUR^AV/AV^ mice generated more myogenic tone and were more sensitive to PE than arteries from WT mice (Supplemental Figure 2, A–D). Myogenic tone was also assessed after endothelial cell function had been disrupted by passing air through the lumen. Following this maneuver, the myogenic tone of mesenteric arteries from Kir6.1^wt/VM^ (Figure 3, E and F) and SUR^AV/AV^ (Supplemental Figure 3) mice did not differ from those of controls. These data suggest that the endothelium exerts a tonic relaxation that opposes myogenic constriction in arteries from control mice but not from Cantú mice. We also found that myogenic tone was elevated in cerebral arteries from Kir6.1^wt/VM^ and SUR^AV/AV^ mice (Supplemental Figure 4), suggesting that multiple vascular beds are similarly affected by Cantú mutations.

In control studies, we found that constriction of mesenteric arteries from Kir6.1^wt/VM^ and SUR^AV/AV^ mice in response to direct depolarization of SMCs by elevated extracellular [K^+^] (60 mM) did not differ from that of controls (Supplemental Figure 5), suggesting that voltage-dependent Ca^2+^ influx and contractile mechanisms are unaffected by K_ATP_ GOF. We also found that the passive diameters of mesenteric arteries from control and Cantú mice did not differ from each other as higher levels of intraluminal pressure were applied, suggesting that K_ATP_ GOF has no effect on vascular compliance (Supplemental Figure 6).

### K_ATP_ channel activation increases endothelial cell Ca^2+^ influx.

Muscarinic agonist-induced vasodilation is associated with dynamic Ca^2+^-signaling activity in the endothelium (20). The effect of K_ATP_ channel activation on CCh-induced Ca^2+^ signaling was investigated using third-order mesenteric arteries from *Cdh5*-GCaMP8 mice expressing a genetically encoded Ca^2+^ biosensor exclusively in the endothelium (28). Isolated arteries were sliced longitudinally, pinned to a Sylgard block en face*,* and imaged using high-speed, high-resolution confocal microscopy, as previously described (29). This preparation allows direct imaging of the intact endothelium but also removes the effects of factors that influence the vascular endothelium, such as intraluminal pressure and shear stress.

**Figure 4:**
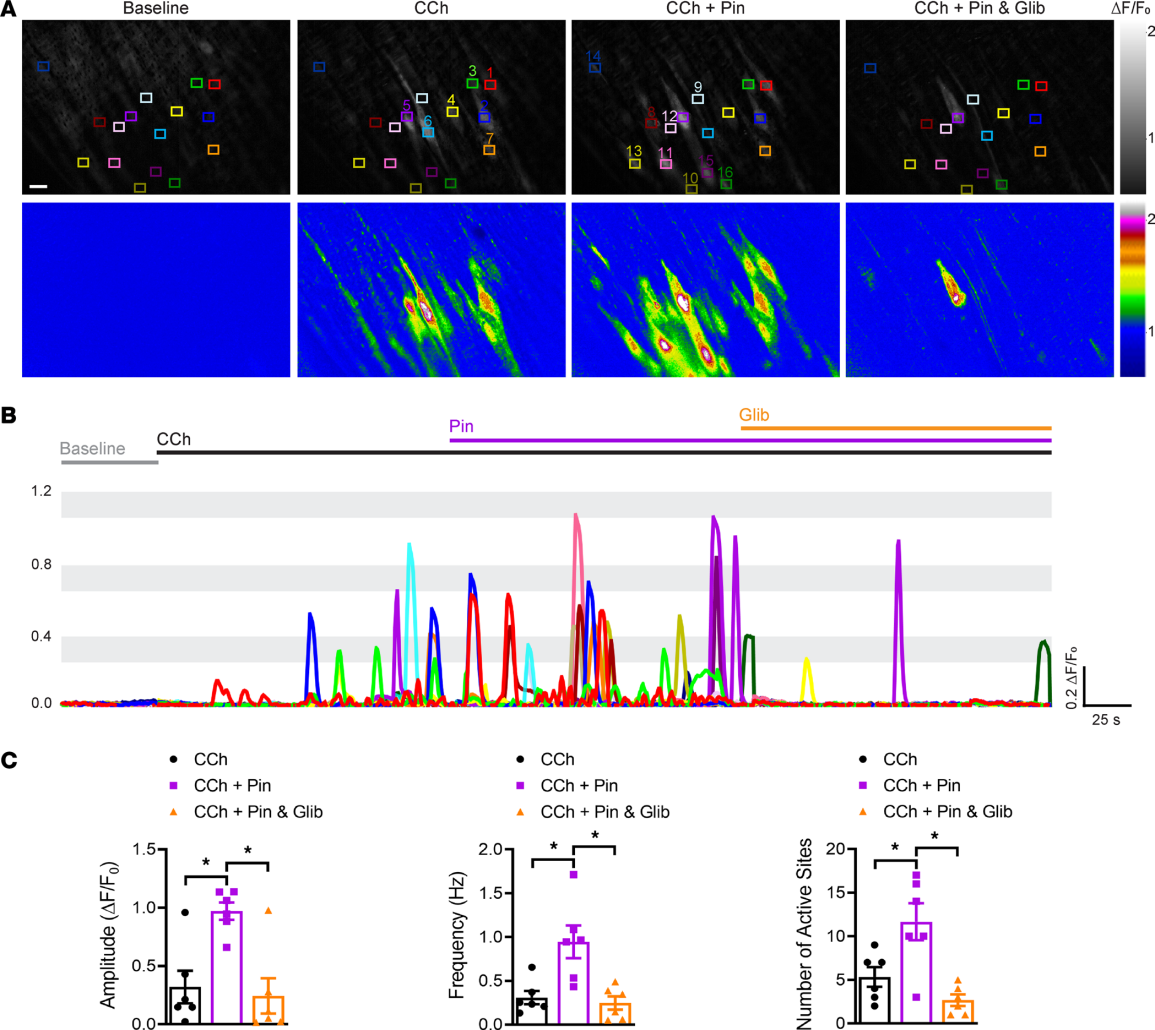
K_ATP_ channel activation increases endothelial cell Ca^2+^ influx. (**A**) Representative grayscale and pseudocolored images of mesenteric arteries from *Cdh5*-GCaMP8 mice mounted en face. Recordings were made initially under baseline conditions for 50 seconds and then in the presence of CCh (10 μM) for 150 seconds, followed by treatment with pinacidil (Pin; 10 μM) for 150 seconds and subsequently with glibenclamide (Glib; 10 μM) in the presence of CCh and pinacidil for 150 seconds. Colored boxes show regions of interest (ROIs) where Ca^2+^ events occurred. The colors indicate the different ROIs that are represented in the time series traces. Scale bar: 10 μm. (**B**) Representative ΔF/F_0_ versus time plots of Ca^2+^ events from multiple Ca^2+^-event sites. (**C**) Summary data showing the effects of pinacidil and glibenclamide on the amplitude (ΔF/F_0_), frequency (Hz), and number of active sites. Data are presented as means ± SEM (*n* = 6 arteries from 3 animals; *P < 0.05, 1-way ANOVA with Tukey’s multiple comparisons test).

The addition of CCh induced Ca^2+^ wave-like events in the endothelium (Figure 4, A and B and Supplemental Video 1). Activation of K_ATP_ channels with pinacidil significantly increased the amplitude, frequency, and number of active sites of CCh-induced Ca^2+^ events (Figure 4C). The increase in Ca^2+^ event frequency was mainly attributable to the recruitment of previously inactive sites (Figure 4C). Blocking K_ATP_ channels with glibenclamide reversed pinacidil-induced increases in Ca^2+^ signaling activity (Figure 4, A–C and Supplemental Video 1). These data indicate that K_ATP_ channel activation augments CCh-induced Ca^2+^ signaling activity in the endothelium.

CCh can orchestrate the release of Ca^2+^ from the ER (20, 30) and promote Ca^2+^ influx through TRPC3, TRPC6 (31, 32), and TRPV4 channels (20). We thus performed additional experiments to probe which of these pathways was affected by K_ATP_ channel activation. We first treated mesenteric arteries with the sarcoplasmic/ER Ca^2+^-ATPase (SERCA) inhibitor thapsigargin (Tg) to deplete ER Ca^2+^ stores. In control cells treated with Tg, CCh induced a few small amplitude Ca^2+^ events (Supplemental Figure 7). The addition of pinacidil significantly increased the amplitude, frequency, and number of active sites, a response that was blunted by glibenclamide (Supplemental Figure 7). These data indicate that when ER Ca^2+^ stores are depleted, increased K_ATP_ channel activity enhances CCh-induced Ca^2+^ signals by promoting Ca^2+^ influx.

Pinacidil did not induce endothelial Ca^2+^ signals in tissue bathed in a Ca^2+^-free solution. Wave-like Ca^2+^ signals were initiated upon introduction of extracellular Ca^2+^ (2 mM), and this effect was significantly diminished by glibenclamide (Supplemental Figure 8, A–C and Supplemental Video 2). These data suggest that activation of K_ATP_ channels stimulates Ca^2+^ influx in the endothelium.

In the absence of extracellular Ca^2+^, CCh initiated low-frequency Ca^2+^-signaling activity. Under these same conditions, adding pinacidil did not change the amplitude or frequency of CCh-induced Ca^2+^ events or number of active sites. Ca^2+^-signaling activity was increased upon restoration of extracellular Ca^2+^, and this effect was blunted by glibenclamide (Supplemental Figure 8, D–F). These data provide evidence that CCh can generate Ca^2+^ signaling activity by releasing intracellular stores and that pharmacological activation of K_ATP_ channels amplifies this effect by promoting endothelial cell Ca^2+^ influx.

### Augmented endothelial cell Ca^2+^ signaling activity in Kir6.1^wt/VM^ mice.

**Figure 5:**
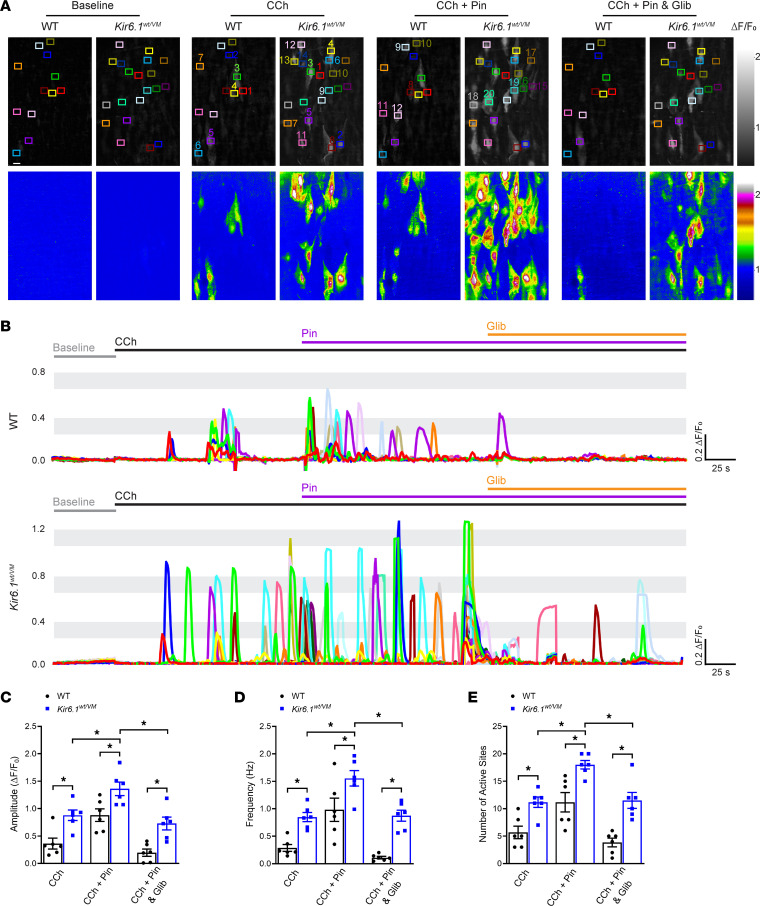
Augmented endothelial cell Ca^2+^ signaling activity in Kir6.1^wt/VM^ mice. (**A**) Representative grayscale and pseudocolored images of mesenteric arteries from *Cdh*5-GCaMP8 and *Cdh*5-GCaMP8 x Kir6.1^wt/VM^ mice mounted en face. Recordings were made initially under baseline conditions for 50 seconds and then in the presence of CCh (10 μM) for 150 seconds, followed by treatment with pinacidil (Pin; 10 μM) for 150 seconds and subsequently with glibenclamide (Glib; 10 μM) in the presence of CCh and pinacidil for 150 seconds. Colored boxes show ROIs with active Ca^2+^ signals. The colors indicate the different ROIs that are represented in the time series traces. Scale bar: 10 μm. (**B**) Representative ΔF/F_0_ versus time plots of Ca^2+^ events from multiple ROIs. (**C–E**) Summary data showing the effects of pinacidil and glibenclamide on the amplitude (ΔF/F_0_), frequency (Hz), and number of active sites. Data are presented as means ± SEM (*n* = 6 arteries from 3 animals per group; *P < 0.05, 2-way ANOVA with Tukey’s multiple comparisons test).

To investigate changes in CCh-induced endothelial cell Ca^2+^ signaling associated with Cantú syndrome, we crossed *Cdh5*-GCaMP8 and Kir6.1^wt/VM^ mice. The amplitude and frequency of CCh-induced Ca^2+^-signaling events and number of active sites were significantly greater in arteries from *Cdh5*-GCaMP8 x Kir6.1^wt/VM^ mice than in those from controls (Figure 5, A–E and Supplemental Video 3). Pinacidil increased the amplitude and frequency of CCh-induced Ca^2+^ events and number of active sites in both groups of animals, but the response was greater in arteries from *Cdh5*-GCaMP8 x Kir6.1^wt/VM^ mice (Figure 5, A–E and Supplemental Video 3). Subsequent application of glibenclamide reduced the effects of pinacidil in both groups (Figure 5, A–E and Supplemental Video 3), but this effect was less prominent in arteries from *Cdh5*-GCaMP8 x Kir6.1^wt/VM^ mice, consistent with the reduced glibenclamide sensitivity of mutant K_ATP_ channels expressed by Kir6.1^wt/VM^ mice (2). These data demonstrate that CCh-induced Ca^2+^-signaling activity is elevated in the endothelium of Kir6.1^wt/VM^ mice.

### Elevated endothelial cell mitochondrial [Ca^2+^] increases ROS generation in Cantú mice.

**Figure 6:**
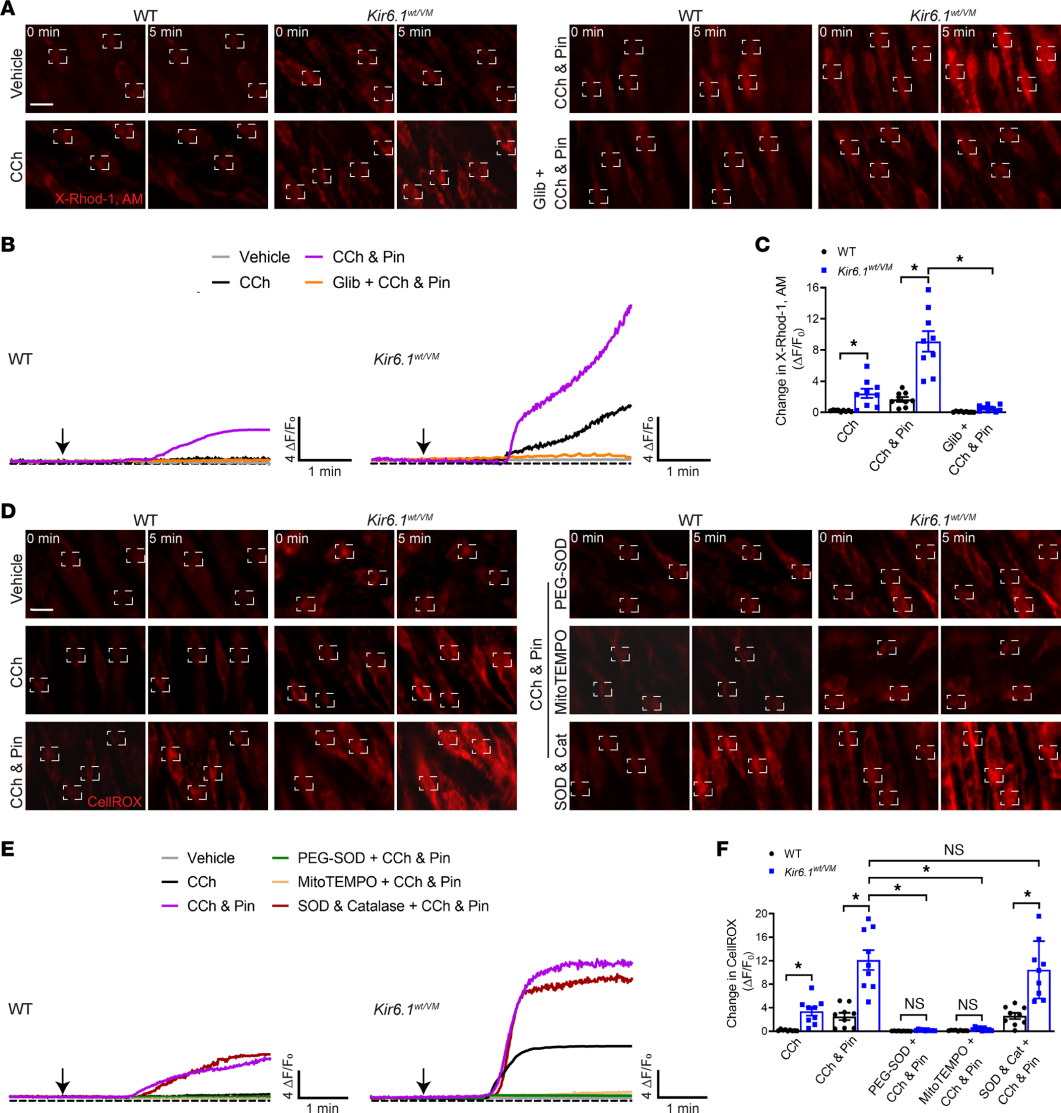
Elevated mitochondrial [Ca^2+^] and increased ROS generation in the endothelium of Cantú mice. (**A**) Representative images of mesenteric arteries from WT and Kir6.1^wt/VM^ mice mounted en face and loaded with the mitochondrial-specific Ca^2+^ indicator X-Rhod-1, AM. Changes in X-Rhod-1 fluorescence were detected following treatment with CCh (10 μM), pinacidil (Pin; 10 μM), and glibenclamide (Glib; 10 μM). Boxes show regions of interest (ROIs). Scale bar: 10 μm. (**B**) Representative ΔF/F_0_ versus time plots of X-Rhod-1 fluorescence for each condition. Arrows indicate application of the agonist. (**C**) Summary data showing changes in X-Rhod-1 fluorescence intensity. Data are presented as means ± SEM (*n* = 9 arteries from 3 animals per group; *P < 0.05, 2-way ANOVA with Tukey’s multiple comparisons test). (**D**) Representative images of mesenteric arteries from WT and Kir6.1^wt/VM^ mice mounted en face and loaded with the superoxidesensitive fluorescent dye CellRox. Changes in CellRox fluorescence were imaged following treatment with CCh, (10 μM), Pin (10 μM), PEG-SOD (100 U/mL), the mitochondrial ROS scavenger mitoTEMPO (5 μM), or a combination of SOD (500 U/mL) and catalase (500 U/mL). Boxes show regions of interest (ROIs). Scale bar: 10 μm. (**E**) Representative ΔF/F_0_ versus time plots of the change in fluorescence intensity under each condition. Arrows indicate application of the agonist. (**F**) Summary data showing changes in CellRox fluorescence intensity. Data are presented as means ± SEM (*n* = 9 arteries from 3 animals per group; *P < 0.05, 2-way ANOVA with Tukey’s multiple comparisons test).

Next, we investigated whether chronically elevated endothelial cell Ca^2+^ influx might be responsible for the loss of endothelium-dependent vasodilation in Kir6.1^wt/VM^ mice. Prior studies have shown that the ER and the mitochondria buffer abnormal cytosolic [Ca^2+^] increases (33). However, elevated mitochondrial [Ca^2+^] can damage the electron transport chain, leading to excessive mitochondrial ROS generation and oxidative stress (34). Oxidative stress, in turn, causes endothelial dysfunction by limiting the bioavailability of ·NO and disrupting other pathways (35–38). To determine whether elevated endothelial cell Ca^2+^ influx in Cantú mice increases mitochondrial [Ca^2+^], we mounted mesenteric arteries from control and Kir6.1^wt/VM^ mice en face and loaded them with the selective mitochondrial Ca^2+^ indicator dye X-Rhod-1-AM (39) (Supplemental Figure 9A). CCh significantly increased mitochondrial [Ca^2+^] in the endothelium of arteries from Kir6.1^wt/VM^ mice but not in those from littermate controls (Figure 6, A–C). Acute application of pinacidil in the presence of CCh resulted in a time-dependent increase in mitochondrial [Ca^2+^] in both groups, an increase that was greater in arteries from Kir6.1^wt/VM^ mice (Figure 6, A–C). Glibenclamide significantly diminished the effects of pinacidil in both groups (Figure 6, A–C). These data indicate that increased CCh-induced Ca^2+^ signaling activity in the endothelium of mesenteric arteries from Cantú mice is accompanied by a rise in mitochondrial [Ca^2+^]. Moreover, this effect was mimicked in controls by pharmacological activation of K_ATP_ channels.

To determine whether elevated mitochondrial [Ca^2+^] was associated with increased generation of ROS, we loaded en face-mounted mesenteric arteries with the mitochondrial ROS probe CellRox (40). Selective loading of mitochondria by CellRox was confirmed by colabeling the endothelium with MitoTracker green (Supplemental Figure 9B). However, our loading and imaging conditions do not completely eliminate signals generated in SMCs. Again, CCh was without effect in arteries from control animals but it significantly increased mitochondrial ROS levels in mesenteric arteries from Kir6.1^wt/VM^ mice (Figure 6, D–F). Following the application of pinacidil, CCh increased mitochondrial ROS in arteries from both WT control and Kir6.1^wt/VM^ mice (Figure 6, D–F). Treatment with membrane-permeant pegylated superoxide dismutase (PEG-SOD) or a mitochondrial-targeted form of the SOD mimetic TEMPO (mitoTEMPO) essentially abolished CCh-induced increases in mitochondrial ROS, whereas combined treatment with membrane-impermeant SOD and catalase had no effect (Figure 6, D–F). These data indicate that either genetic or pharmacologically induced activation of K_ATP_ channels causes an increase in mitochondrial ROS generation in the endothelium following CCh stimulation.

To determine whether mitochondrial ROS generation also elevated cytosolic ROS levels in the endothelium of Cantú mice, we loaded en face-mounted mesenteric arteries with the global ROS indicator dye CM-H2DCFDA. CM-H2DCFDA passively diffuses into cells, where its acetate groups are cleaved by intracellular esterases and its thiol-reactive chloromethyl group reacts with intracellular glutathione and other thiols. Subsequent oxidation yields a fluorescent adduct (41). CCh had no effect on vessels from control animals but markedly elevated ROS levels in arteries obtained from Kir6.1^wt/VM^ mice. Subsequent application of pinacidil further increased ROS in both control and Kir6.1^wt/VM^ mice (Supplemental Figure 10). Moreover, treatment with mitoTEMPO essentially eliminated CCh-induced increases in ROS (Supplemental Figure 10). These data suggest that mitochondrial ROS generation increases global ROS levels in the endothelium of K_ATP_ GOF mice.

### Elevated ONOO^–^ levels in the endothelium of Cantú mice.

**Figure 7:**
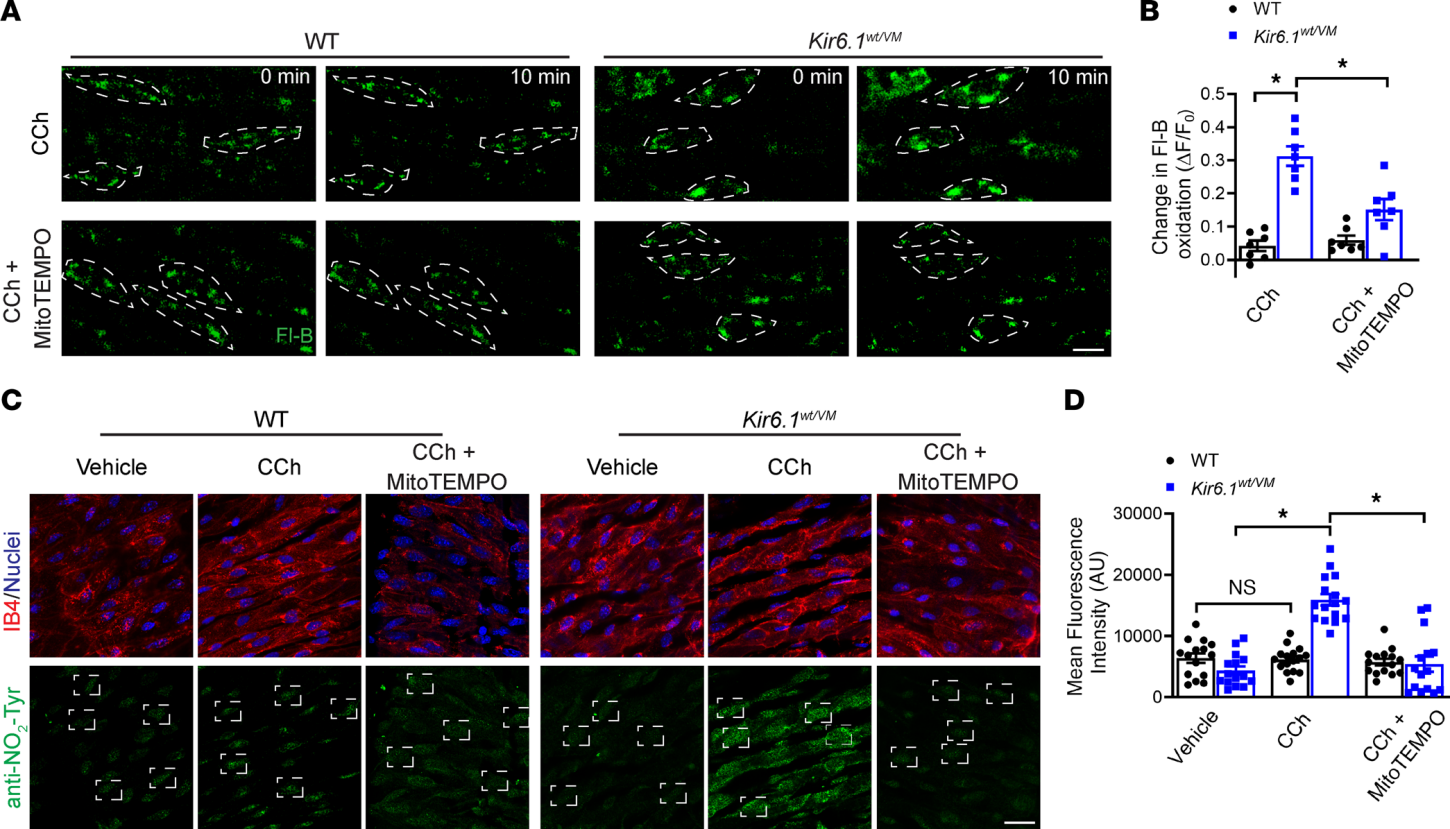
ONOO^–^ levels are increased in the endothelium of Cantú mice. (**A)** Representative images of mesenteric arteries from WT and Kir6.1^wt/VM^ mice mounted en face and loaded with fluorescein-boronate (Fl-B) and treated with CCh (10 μM) or CCh and mitoTEMPO (5 μM). The dashed squares show individual endothelial cells. Scale bar: 10 μm (**B**) Summary data showing the change in fluorescence intensity upon Fl-B oxidation± SEM. *n* = 7 arteries from 3 animals per group, *P < 0.05, 2-way ANOVA with Tukey’s multiple comparisons test. (**C**) Representative images of mesenteric arteries from WT and Kir6.1^wt/VM^ mice mounted en face and labeled with IB4 (red) and DAPI (blue). The bottom panels show immunolabeling of nitrated tyrosine residues (green). Boxes show regions of interest (ROIs). Scale bar: 20 μm. (**D**) Summary data showing mean fluorescent intensity ± SEM for nitrated tyrosine signal. *n* = 15 cells from 3 animals per group, *P < 0.05, 2-way ANOVA with Tukey’s multiple comparisons test.

Superoxide radicals (O_2_·^–^) rapidly react with endothelium-derived ·NO to form ONOO^–^ (42–44), a process that impairs endothelium-dependent vasodilation by reducing the bioavailability of ·NO. ONOO^–^ can also directly affect Ca^2+^ influx through TRPV4 channels and subsequent activation of small- and intermediate-conductance Ca^2+^-activated K^+^ channels (SK and IK, respectively) (36). To determine whether the elevated mitochondrial ROS levels associated with K_ATP_ GOF affected the production of ONOO^–^ in the endothelium, we loaded mesenteric arteries with fluorescein-boronate (Fl-B), a fluorescent probe used to detect ONOO^–^ (45). CCh induced an increase in fluorescence in arteries from Kir6.1^wt/VM^ mice but not from WT controls, and this response was blunted by pretreatment of arteries with mitoTEMPO (Figure 7, A and B). These data suggest that elevated mitochondrial ROS generation increases ONOO^–^ production in the endothelium of arteries of Kir6.1^wt/VM^ mice. Elevated ONOO^–^ increases the nitration of tyrosine (Tyr) residues (46, 47). We performed immunostaining using an anti-nitrotyrosine (NO_2_-Tyr) antibody to measure Tyr nitration in mesenteric arteries. Treatment with CCh increased NO_2_-Tyr levels in arteries from Kir6.1^wt/VM^ mice but not in WT controls (Figure 7, C and D). Further, pretreatment with mitoTEMPO diminished CCh-induced increases in NO_2_-Tyr levels in arteries from Kir6.1^wt/VM^ mice (Figure 7, C and D). These data provide additional evidence that ONOO^–^ levels are elevated in mesenteric arteries from Kir6.1^wt/VM^ mice.

### Mitochondrial ROS generation impairs endothelium-dependent vasodilation in Cantú mice.

**Figure 8:**
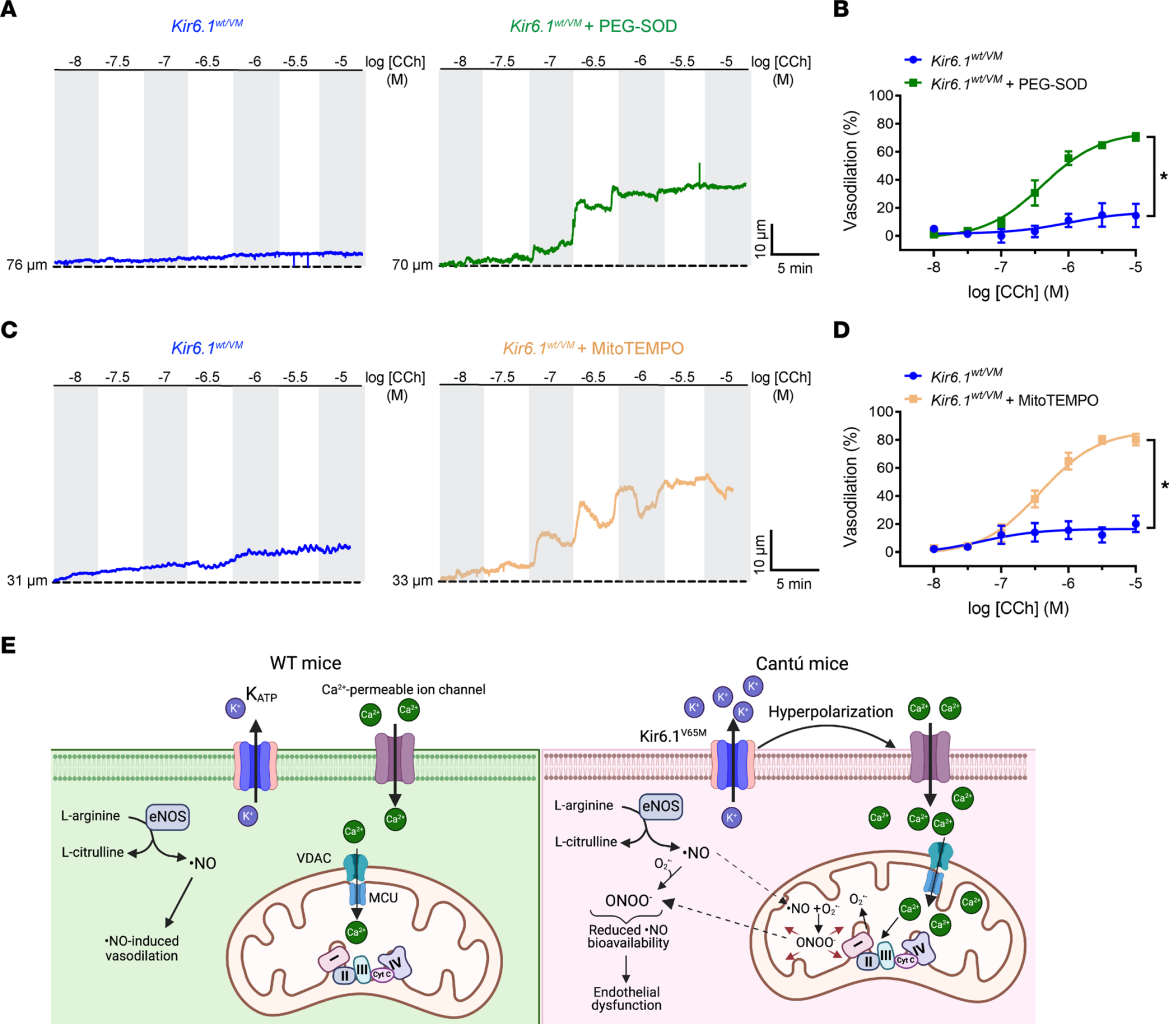
Mitochondrial ROS generation impairs endothelium-dependent vasodilation in Cantú mice. (**A**) Representative recordings and (**B**) summary data showing CCh-evoked, endothelium-dependent dilation of mesenteric arteries from Kir6.1^wt/VM^ mice before and after treatment with the membrane-permeable intracellular ROS scavenger PEG-SOD (100 U/mL). Data are presented as means ± SEM (*n* = 5 vessels from 4 animals per group; *P < 0.05, 2-way ANOVA with Šídák’s multiple comparisons test). (**C**) Representative recordings and (**D**) summary data showing CCh-evoked, endothelium-dependent dilation of mesenteric arteries from Kir6.1^wt/VM^ mice before and after treatment with the mitochondria-targeted superoxide mimetic mitoTEMPO (5 μM). Data are presented as means ± SEM (*n* = 6 vessels from 4 animals per group; *P < 0.05, 2-way ANOVA with Šídák’s multiple comparisons test). (**E**) Schematic depicting the proposed model for endothelial dysfunction associated with Cantú syndrome. Endothelial cell K_ATP_ GOF hyperpolarizes the plasma membrane, increasing the electrochemical driving force for Ca^2+^ influx. Elevated cytosolic Ca^2+^ levels are partially buffered by the mitochondria, leading to increased ROS generation. Elevated mitochondrial ROS levels reduce ·NO bioavailability and generate ONOO^–^ in situ, impairing endothelial-dependent vasodilation. Secondarily, disruption of mitochondrial and cell homeostasis promotes cytosolic ONOO- formation and further affects vascular function and integrity (44, 47, 72).

To determine if impaired endothelium-dependent dilation of mesenteric arteries from Kir6.1^wt/VM^ mice is caused by increased mitochondrial ROS generation, we used ROS scavengers. PEG-SOD fully restored endothelium-dependent dilation in arteries from Kir6.1^wt/VM^ mice (Figure 8, A and B), suggesting that increased intracellular ROS generation is responsible for endothelium dysfunction in Cantú mice. Combined administration of plasma membrane–impermeant forms of SOD and catalase did not improve endothelium-dependent dilation of arteries from Kir6.1^wt/VM^ mice (Supplemental Figure 11), suggesting no contribution of extracellular ROS generation to dysfunction. We also found that blocking mitochondrial ROS with mitoTEMPO rescued endothelium-dependent vasodilation in Kir6.1^wt/VM^ mice (Figure 8, C and D). In addition, the contractility of arteries from Kir6.1^wt/VM^ mice was restored to control levels by blocking the effects of mitochondrial ROS with mitoTEMPO (Supplemental Figure 12). These data suggest that excessive mitochondrial ROS generation is responsible for endothelial dysfunction and associated arterial hypercontractility in Cantú syndrome.

We propose the following model for endothelial dysfunction in Cantú syndrome (Figure 8E): K_ATP_ overactivity hyperpolarizes the endothelial cell plasma membrane, driving endothelial cell Ca^2+^ influx. Excessive cytosolic Ca^2+^ levels are buffered by mitochondria, leading to increased ROS generation. Elevated ROS levels, in turn, cause endothelial dysfunction by decreasing the bioavailability of ·NO and/or increasing ONOO^–^ production.

## Discussion

Autosomal dominant GOF mutations in *KCNJ8* and *ABCC9,* encoding Kir6.1 and SUR2, respectively, cause Cantú syndrome and associated cardiovascular abnormalities (1, 2, 5, 48). Massively increased heart size and elevated cardiac output are key features of the disease (49, 50). Prior studies have focused on the effect of Cantú syndrome on vascular SMCs and have shown that K_ATP_ overactivity in these cells is associated with lowered blood pressure and secondarily causes cardiac enlargement (2, 23). However, Kir6.1 and SUR2B are also prominently expressed in vascular endothelial cells (1, 13, 14), but the effects of K_ATP_ GOF mutations in this cell type and their role in the manifestations of Cantú syndrome have not been previously investigated. Here, we show that elevated K_ATP_ channel activity in native endothelial cells from Cantú mice hyperpolarizes the resting membrane potential. Further, endothelium-dependent vasodilation is nearly absent in small mesenteric arteries from Cantú mice, resulting in arterial hypercontractility. Vasodilator agonist-induced Ca^2+^ influx and Ca^2+^-signaling activity are elevated in the endothelium of Cantú mice, likely driven by K_ATP_-induced plasma membrane hyperpolarization. K_ATP_ GOF mutations also increase mitochondrial [Ca^2+^] and ROS generation in the endothelium in association with increased levels of the ·NO metabolite ONOO^–^. We propose that diminished bioavailability of ·NO and/or direct effects of ONOO^–^ on ·NO-independent vasodilator pathways are responsible for endothelial dysfunction in K_ATP_ GOF mutants. Our findings describe endothelial dysfunction as a pathological facet of Cantú syndrome.

K_ATP_ channel activity is low when ATP is abundant and increases when ATP levels decrease and/or ADP levels increase (51–53). Thus, K_ATP_ channels are a molecular link between cellular metabolic state and membrane excitability (51–54). Kir6.1/SUR2B–dependent K_ATP_ channels are highly expressed throughout the vasculature, and their activation in SMCs hyperpolarizes the plasma membrane, thereby decreasing voltage-dependent Ca^2+^ influx and diminishing contractility (2). In contrast, in the electrically unexcitable endothelium, K_ATP_ channel activity hyperpolarizes the membrane, increasing the electrochemical driving force for Ca^2+^ influx via voltage-independent pathways (14, 21). This potentially acts as an adaptive mechanism that enhances Ca^2+^-dependent vasodilator pathways, increasing blood flow and nutrient delivery when ATP production is diminished (13, 53, 55). Our data show that agonist-induced Ca^2+^ influx is enhanced in the endothelium of Kir6.1 GOF mutants due to a hyperpolarized resting membrane potential. However, rather than strengthening endothelium-dependent dilation, our data suggest that enhanced agonist-induced Ca^2+^-signaling activity is associated with elevated mitochondrial Ca^2+^ and ROS levels and endothelial dysfunction.

Prior studies have shown that mitochondria can buffer pathological increases in cytosolic [Ca^2+^] through uptake mediated by the mitochondrial Ca^2+^ uniporter (MCU) (56–58) and/or the mitochondrial Na^+^/Ca^2+^ exchanger (NCLX) operating in reverse mode. Either or both mechanisms could be responsible for the increases in mitochondrial [Ca^2+^] we report. Excess mitochondrial [Ca^2+^] has been linked to several diseases, including heart failure and Alzheimer’s disease (59–61). Hyperuricemia causes NCLX-dependent mitochondrial Ca^2+^ overload and elevated ROS generation, leading to diminished ·NO production (62). Other studies have shown that head and neck irradiation elevates mitochondrial [Ca^2+^] and ROS generation in endothelial cells and causes endothelial dysfunction in mice (63). This effect is prevented by MCU KO in endothelial cells (63), suggesting that mitochondrial Ca^2+^ uptake is ultimately responsible for the pathology. Our data indicate that chronic overactivity of K_ATP_ channels also produces deleterious effects on the vascular endothelium by driving increases in mitochondrial Ca^2+^ uptake and ROS generation. Although K_ATP_ channels are reported to be present in the mitochondrial membrane (64), their activity would be expected to depolarize the mitochondrial membrane and reduce the driving force for Ca^2+^ uptake (65). Thus, it is likely that enhanced cytosolic Ca^2+^ influx resulting from endothelial cell plasma membrane hyperpolarization is responsible for driving mitochondrial Ca^2+^ overload in Cantú mice.

Endothelial dysfunction, characterized by the loss of endogenous vasodilator pathways, is a hallmark of atherosclerosis, hypertension, and other common cardiovascular diseases (66, 67). Inflammation, hypercholesterolemia, obesity, aging, and multiple other factors can lead to the downregulation of endothelial ·NO synthase (eNOS) expression (68) or dysregulated phosphorylation of specific amino acids during cardiovascular disease (69), ultimately leading to decreased ·NO production and impaired endothelium-dependent vasodilation (36, 70, 71). Endothelial dysfunction is caused by oxidative stress through several mechanisms, including diminished bioavailability of ·NO through conversion to ONOO^–^ (72). Here, we provide evidence that elevated ROS levels cause endothelial dysfunction in K_ATP_ GOF mice through this process, demonstrating the pathological consequences of chronic channel overactivity. Interestingly, another study showed that endothelial cell K_ATP_ knockout exacerbates endothelial dysfunction, hypertension, and atherosclerosis induced by high-salt and high-fat diets (16), suggesting that, when operating normally, the channel provides protection against these cardiovascular diseases. We conclude that vascular function relies on appropriate modulation of endothelial cell K_ATP_ channels.

Approximately 23% (16 of 71) of the patients enrolled in a Cantú registry reported blood pressure irregularities. Of this subset, 62.5% (10 of 16) were hypertensive, and 37.5% (6 of 16) were clinically hypotensive (10). This inconsistency could reflect the effects of different mutations on K_ATP_ channel function or the impact of age or other environmental and genetic factors. A prior study indicated that the K_ATP_ GOF mice used here are hypotensive because of decreased systemic vascular resistance (2). However, our findings show that, despite elevated K_ATP_ channel activity in SMCs, small mesenteric resistance arteries from Kir6.1^wt/VM^ and SUR^AV/AV^ mice become hypercontractile because the endothelium is dysfunctional. We propose that, during Cantú syndrome, endothelial dysfunction is a coincident adaptation that moderates the vasodilatory effects of tonic SMC hyperpolarization, thereby preventing life-threatening hypotension. Decreases in systemic vascular resistance reported for K_ATP_ GOF mice could occur at the level of the capillaries. The contractile pericytes that encircle capillaries express high levels of K_ATP_ channels, which regulate capillary diameter and microvascular blood flow in response to metabolic cues (13, 55). K_ATP_ activation is predicted to cause pericyte relaxation and promote capillary dilation, thereby decreasing systemic vascular resistance and blood pressure.

In summary, we have identified mechanistic consequences of K_ATP_ overactivity in the endothelium of Cantú syndrome mice. This overactivity leads to Ca^2+^ overload, mitochondrial dysfunction, and ROS generation, which subsequently leads to ·NO consumption and ONOO^–^ formation, removing the tonic vasorelaxant effect of the endothelium on the underlying vascular SMCs. This counteracts the intrinsically hypotensive effect of chronically elevated K_ATP_ channel activity in SMCs and serves as a coincident mechanism that may help to maintain blood pressure at an adequate level to support tissue perfusion. Although our study identifies endothelial dysfunction as a pathological aspect of Cantú syndrome, translating these findings into clinical interventions requires further validation.

## Methods

### Sex as a biological variant.

Adult (3–4 month old) male and female mice were used for all investigations. Sex-related differences in the data presented here were not observed.

### Animals.

Animals were housed under controlled 12-hour light–dark cycles in individually ventilated cages (≤ 5 mice/cage) with unrestricted access to food and water. The generation of Cantú mice was reported previously by Huang et al. (2). CRISPR/Cas9–mediated genome editing was employed to generate mice carrying mutations equivalent to CS-causing human GOF mutations in *KCNJ8* or *ABCC9* genes. Heterozygous Kir6.1 [V65M] and homozygous SUR2 [A476V] (SUR2 [A478V] in the human sequence) mutant mice were used for this investigation. *Cdh5*-GCaMP8 transgenic mice, generated as part of the Cornell/National Heart Lung Blood Resource for Optogenetic Mouse Signaling (CHROMus) at Cornell University (73) (Jackson Labs Stock number 033342), provide an excellent signal-to-noise ratio for optical detection of Ca^2+^ events in the endothelium of mesenteric arteries (28). Heterozygous Kir6.1^wt/VM^ mice were crossed with *Cdh5*-GCaMP8 mice to study Ca^2+^ signaling in the endothelium of K_ATP_ GOF mice. Founder animals were identified by PCR-based genotyping (to detect *Cdh5*-GCaMP8) and genomic DNA sequencing (to detect the missense mutation in Kir6.1^wt/VM^). mT/mG mice (Jackson Labs, stock number: 007676) (24) were crossed with mice expressing Cre-recombinase under control of the *Cdh5* promoter (*Vec^cre^*) to label endothelial cells with eGFP.

Mice were euthanized under isoflurane anesthesia (Baxter Healthcare) by decapitation and exsanguination. The mesentery was isolated and immediately placed in an ice-cold Ca^2+^-free physiological saline solution (Mg-PSS; containing 140 mM NaCl, 5 mM KCl, 2 mM MgCl_2_, 10 mM HEPES, and 10 mM glucose (pH 7.4), supplemented with 0.5% BSA). The buffer was changed as necessary to remove released fecal material.

### Pressure myography.

Pressure myography studies were conducted in accordance with recent best-practice recommendations (27). Isolated mouse mesenteries were gently pinned in a Sylgard-coated Petri dish containing Ca^2+^-free Mg-PSS supplemented with 0.5% BSA. Third-order arteries were carefully dissected, cannulated between 2 glass cannulas (outer diameter, approximately 40–50 μm) in a pressure myograph chamber (Living Systems Instrumentation), and securely tied with a nylon thread. An inverted microscope (Accu-Scope Inc.) connected to a USB camera (The Imaging Source LLC) was used to visualize arteries. IonWizard software (version 7.2.7.138; IonOptix LLC) was used to measure changes in vessel luminal diameter. Preparations were kept in warmed (37°C), oxygenated (21% O_2_, 6% CO_2_, 73% N_2_) PSS (119 mM NaCl, 4.7 mM KCl, 21 mM NaHCO_3_, 1.17 mM MgSO_4_, 1.8 mM CaCl_2_, 1.18 mM KH_2_PO_4_, 5 mM glucose, 0.03 mM EDTA) at an intraluminal pressure of 5 mmHg for 15 minutes. Arteries were then pressurized to 110 mmHg and stretched to their approximate in vivo length, and the pressure was returned to 5 mmHg for 15 minutes. The viability of each vessel was evaluated by measuring vasoconstrictor responses to high extracellular [K^+^] PSS (60 mM KCl, 63.7 mM NaCl). Arteries that constricted by less than 10% when treated with high [K^+^] PSS were excluded from further study (27).

Myogenic tone was assessed by gradually raising intraluminal pressure from 5 mmHg to 20 mmHg and subsequently to 100 mmHg in increments of 20 mmHg. At each pressure, vessels were allowed to develop spontaneous tone until a steady state diameter was reached. After recording the active diameter, intraluminal pressure was dropped to 5 mmHg, and arteries were bathed with Ca^2+^-free PSS supplemented with the Ca^2+^ chelator EGTA (2 mM) and the voltage-dependent Ca^2+^ channel blocker diltiazem (10 μM). The passive diameter was then determined at each pressure step. Myogenic tone (%) was calculated as [1 – (active luminal diameter/passive luminal diameter)] x 100. In additional studies, vasoconstriction in response to increasing concentrations of phenylephrine (PE) was measured using arteries pressurized to 20 mmHg. In some studies, the endothelium was disrupted by passing air bubbles through the vessel lumen, as previously described (74).

To assess endothelium-dependent vasodilation, vessels were pressurized to 20 mmHg and preconstructed by application of U-46619 (Cayman Chemical, 100 nM) to establish a consistent level of vascular tone across groups. The change in luminal diameter was recorded in response to increasing concentrations of the muscarinic agonist CCh. Vasodilation was calculated as the CCh-induced change in diameter (maximum diameter in the presence of CCh – basal diameter) normalized to the vessel’s passive diameter at each concentration, i.e., Vasodilation (%) = (change in diameter/(passive diameter – basal diameter) ×100.

### Ca^2+^ imaging and analysis.

Ca^2+^ signals were recorded in third-order mesenteric arteries isolated from *Cdh5*-GCaMP8 and *Cdh5*-GCaMP8 x Kir6.1^wt/VM^ mice. As previously described (28, 29), isolated arteries bathed in Ca^2+^-free Mg-PSS supplemented with 0.5% BSA were opened longitudinally using fine spring scissors (Fine Science Tools) and mounted en face on a Sylgard pad using insect pins. The tissue was stretched to its in vivo length and maintained in Ca^2+^-free PSS at 37°C for 10 minutes and then superfused with PSS containing 2 mM CaCl_2_ in the presence of CCh (10 μM). In some experiments, arteries were treated with the K_ATP_ channel activator pinacidil (10 μM; Cayman Chemical) and/or the K_ATP_ channel blocker glibenclamide (10 μM; Tocris Bioscience). The solution was continuously bubbled with a normoxic gas mixture (21% O_2_, 6% CO_2_, 73% N_2_) to maintain constant pH and oxygenation. Ca^2+^ images were recorded using a fixed-stage, upright microscope (Olympus, BX51WI). The images were recorded using epifluorescent illumination (CoolLED, PE-300^white^, CoolLED Limited), an eGFP filter set (39002 AT, Chroma Scientific) and an ORCA-Fusion Digital CMOS Camera (C14440-20UP, Hamamatsu). The images were obtained using a 60× water immersion objective at 2 frames/second. Open-source MicroManager software on a Windows PC provided system control and data acquisition.

Ca^2+^ recordings were analyzed using custom-designed SparkAn software (created by Adrian Bonev and provided by Mark T. Nelson, University of Vermont [Burlington, Vermont, USA]) (75). Baseline fluorescence (F_0_) was defined using the first 10 images of each recording captured before stimulation. After subtraction of F_0_, localized increases in fluorescence (ΔF) within a region of interest (ROI) (3′ 3 μm^2^) were identified as Ca^2+^ events. These signals appear as events with distinctive peaks on plots of fluorescence intensity over time. ΔF/F_0_ versus time plots of Ca^2+^ events were obtained from multiple sites for each recording, and amplitude (ΔF/F_0_) was determined individually for each Ca^2+^ peak. The frequency (Hz) and number of active sites were determined for the entire image field.

### Mitochondrial Ca^2+^, ROS, and ONOO^–^ imaging.

The en face mesenteric artery preparation was also used to investigate mitochondrial [Ca^2+^] and ROS levels. Mitochondrial [Ca^2+^] was measured by loading arteries with the mitochondrial Ca^2+^ indicator dye X-Rhod-1-AM (5 μM; Thermo Fisher Scientific) for 30 minutes at room temperature. For mitochondrial ROS measurements, vessels were loaded with the superoxide indicator CellROX Deep Red (5 μM; Thermo Fisher Scientific) for 30 minutes at room temperature. For global intracellular ROS measurements, vessels were incubated with CM-H_2_DCFDA (10 μM; Thermo Fisher Scientific) for 30 minutes at room temperature. Excess dye remaining after loading was eliminated by washing the tissue 3 times, and the chamber was transferred to a confocal microscope for real-time recordings of fluorescence intensity. Recordings were performed using a fixed-stage, upright microscope (Olympus, BX51WI) equipped with an ORCA-Fusion Digital CMOS Camera (Hamamatsu) and epifluorescent illumination (CoolLED, PE-300^white^). Tissues were illuminated using an mCherry filter set for X-Rhod-1 (39010 AT, Chroma Scientific), a Cy5 filter set for CellROX Deep Red (49006 ET, Chroma Scientific), and an eGFP filter set for CM-H_2_DCFDA (39002 AT, Chroma Scientific). Images were acquired using a 60× water-immersion objective at a rate of 1 frame/second for 5 minutes. For pharmacological interventions, tissues were preincubated with the indicated agent (glibenclamide, SOD and catalase, PEG-SOD, and mitoTEMPO) for 15 minutes and then continuously exposed to the agent via the superfusing bath solution throughout the experiment. For some experiments, isolectin-IB4 staining was used to selectively identify endothelial cells in mesenteric arteries. Signal intensities were calculated using Image J (NIH; http://www.Imagej.nih.gov/ij/, RRID: SCR_003070). ΔF/F_0_ versus time plots of fluorescence signals were determined for each recording.

To image ONOO^–^ production vessels were loaded with fluorescein-boronate (Fl-B) (45, 72) (1 μM) for 15 minutes at room temperature. Signals were recorded using a fixed-stage upright microscope (Olympus, BX51WI) equipped with an Andor iXon DU-897 cooled CCD camera (Oxford Instruments). The field of view is 512 × 512 pixels. Illumination was provided by a CSU-X1 Nipkow spinning-disk confocal scanner unit (Yokogawa) and a solid-state laser launch and integrated AOTF. Tissues were excited at 488 nm, and images were acquired using a 60× water immersion objective at 1 frame per 2 minutes for 10 minutes.

### Patch-clamp electrophysiology.

Individual vascular SMCs from mesenteric vessels were isolated as previously described (23). Arteries were gently removed from the mesentery and washed in MgPSS. Native SMCs were obtained by initially digesting isolated arteries in 1 mg/mL papain (Worthington Biochemical Corporation), 1 mg/mL dithiothreitol (DTT), and 10 mg/mL BSA in Mg-PSS at 37°C for 10 minutes, followed by a 10 minute incubation with 1 mg/mL type II collagenase (Worthington Biochemical Corporation). For endothelial cell isolation, arteries were enzymatically digested in 0.5 mg/mL neutral protease and 0.5 mg/mL elastase (Worthington Biochemical Corporation, USA) for 10 minutes at 37°C, followed by a 5 minute incubation with 1 mg/mL type I collagenase (Worthington Biochemical Corporation). A single-cell suspension was prepared by gently washing the digested arteries 3 times and triturating with a fire-polished glass pipette. All cells used for this study were freshly dissociated on the day of experimentation.

Whole-cell K_ATP_ currents were recorded as previously described (2) using an Axopatch 200B amplifier and Digidata 1440A (Molecular Devices). Pipettes were fabricated from borosilicate glass (1.5 mm OD, 1.17 mm ID; Sutter Instruments) and fire-polished to yield a tip resistance of 3–5 MΩ. Recordings were sampled at 3 kHz and filtered at 1 kHz. Currents were initially measured at a holding potential of –70 mV in a high-Na^+^ bath solution containing 136 mM NaCl, 6 mM KCl, 2 mM CaCl_2_, 1 mM MgCl_2_, 10 mM HEPES,and 10 mM glucose, with pH adjusted to 7.4 with NaOH before switching to a high-K^+^ bath solution (140 mM KCl, 2 mM CaCl_2_, 1 mM MgCl_2_, 10 mM HEPES, and 10 mM glucose, with pH adjusted to 7.4 with KOH) in the absence and presence of pinacidil (30 μM) and glibenclamide (30 μM). The pipette solution contained 110 mM K^+^ aspartate, 30 mM KCl, 10 mM NaCl, 1 mM MgCl_2_, 10 mM HEPES, 0.5 mM CaCl_2_, 4 mM K_2_HPO_4_, and 5 mM EGTA, with pH adjusted to 7.2 with KOH. The pipette solution for current-clamp recordings was the same as that used for voltage-clamp experiments. The bath solution was 136 mM NaCl, 2 mM CaCl_2_, 6 mM KCl, 1 mM MgCl_2_, 10 mM HEPES, and 10 mM glucose, with pH adjusted to 7.4 with KOH. Cell-attach patch-clamp experiments used the voltage-clamp configuration to get a gigaseal (>2 GΩ), and then command was switched to current-clamp mode for membrane potential recording. Clampex and Clampfit software (pClamp version 10.7; Molecular Devices) were used for data acquisition and analysis.

### Immunocytochemistry.

Immunofluorescence was used to detect nitrosylated tyrosine residues in the endothelium of mesenteric arteries. Third-order mesenteric arteries were mounted en face, treated with either CCh (10 μM) or CCh and mitoTEMPO, and fixed with 4% formaldehyde in PBS, pH 7.4. Vessels were then washed 3 times with PBS and blocked with 10% SEABlock Blocking Buffer (Thermo Fisher Scientific) and 1% Triton-X in PBS for 2 hours at RT. The arteries were incubated overnight at 4°C with a polyclonal rabbit antinitrotyrosine antibody (1:250; Thermo Fisher Scientific (catalog A-21285) RRID: AB_221457), washed, and incubated with AlexaFluor 568 conjugated isolectin B4 (1:1,000; catalog I21412, Thermo Fisher Scientific), and Alexa Fluor 647 conjugated goat anti-rabbit (catalog A-21244, Life Technologies) for 2 hours at RT. The vessels were then washed 3 times with PBS and incubated with DAPI nuclear staining in PBS, washed again, and mounted on a coverslip using ProLong Gold Antifade Mountant (catalog P36930, Thermo Fisher Scientific). Images were obtained using a Leica Stellaris 5 confocal system, which consists of a DMi8 inverted automated fluorescence microscope (Leica) equipped with solid-state diode lasers (405, 448, 561,and 638 nm). Images were acquired using a 63x/NA 1.40 oil immersion objective with a digital zoom of 1.72. LASX software on a Windows PC provided system control and data acquisition. Images were analyzed using Image J (NIH; http://www.Imagej.nih.gov/ij/, RRID: SCR_003070). Mean fluorescence intensity was measured and reported.

### Chemicals.

Unless expressly stated, all chemicals and reagents used in the study were obtained from Sigma-Aldrich Inc.

### Statistics.

GraphPad Prism software (Version 9.4.1) was used to conduct statistical analyses and generate graphs. The data presented are expressed as means ± SEM, with “n” indicating the number of vessels or cells analyzed. 1-way ANOVA with Tukey’s multiple comparisons test and 2-way ANOVA with Tukey’s or Šídák’s multiple comparisons test were used for statistical analyses. *P* values under 0.05 were considered statistically significant for all analyses.

### Study approval.

All animal care and procedures used in this study were approved by the Institutional Animal Care and Use Committee (IACUC) of the University of Nevada, Reno, School of Medicine (protocol number: 20-06-1020), and are in accordance with the National Institutes of Health (NIH).

### Data availability.

The manuscript and the Supplemental Materials present all the data needed to evaluate the study’s conclusions. All data sets are available on the Dryad platform (https://doi.org/10.5061/dryad.6djh9w19c). All original data sets and analyses, including individual comparisons, are also reported in the Supporting Data Values file.

## Author contributions

SE and CGN initiated and supervised the project. SE and EM designed the experiments. YFE helped develop and manage the transgenic mice used for this study. ASS performed and analyzed patch-clamp electrophysiology experiments. PT, EY, and EM performed pressure myography experiments. EM and BL performed Ca^2+^, ROS, and ONOO^–^ imaging experiments. NR and RR synthesized and provided the ONOO^–^ imaging probe. EM, EY, CM, BL, CGN, and SE analyzed the data. EM and SE wrote the initial manuscript draft and prepared the figures. SE, EM, BL, ASS, CM, RR, NR, YFE, and CGN revised and approved the manuscript.

## Supplementary Material

Supplemental data

Supplemental video 1

Supplemental video 2

Supplemental video 3

Supporting data values

## Version 1 08/01/2024

In-Press Preview

## Version 2 09/10/2024

Electronic publication
