# Supplementary material for: Mitochondrial Ca2+-coupled generation of reactive oxygen species, peroxynitrite formation, and endothelial dysfunction in Cantú syndrome
Source: JCI Insight. 2024 Aug 1;9(17):e176212. doi: 10.1172/jci.insight.176212 (PMC11385080; doi:10.1172/jci.insight.176212)
Supplement: Supplemental data [file jciinsight-9-176212-s159.pdf]

Supplemental Data for:

Mitochondrial Ca^{2+}-coupled generation of reactive oxygen species, peroxynitrite formation, and endothelial dysfunction in Cantú syndrome

Elsayed Metwally[1,2], Alfredo Sanchez Solano[1], Boris Lavanderos[1], Evan Yamasaki[1], Pratish Thakore, Conor McClenaghan[3], Natalia Rios[4,5], Rafael Radi[4,5], Yumei Feng Earley[1], Colin G. Nichols[6], and Scott Earley[1*]

[1]Department of Pharmacology, Center for Molecular and Cellular Signaling in the Cardiovascular System, University of Nevada, Reno School of Medicine, Reno, NV 89557-0318, USA

[2]Department of Cytology and Histology, Faculty of Veterinary Medicine, Suez Canal University, Ismailia, Egypt

[3]Departments of Pharmacology and Medicine, Center for Advanced Biotechnology and Medicine, Robert Wood Johnson Medical School, Rutgers University, Piscataway, NJ 08854, USA

[4]Departamento de Bioquímica, Facultad de Medicina, Centro de Investigaciones Biomédicas, Universidad de la República, Montevideo, Uruguay

[5]Centro de Investigaciones Biomédicas (CEINBIO), Facultad de Medicina, Universidad de la República, Montevideo, Uruguay

[6]Center for the Investigation of Membrane Excitability Diseases, and Departments of Cell Biology and Physiology, Washington University School of Medicine, St. Louis, MO, USA

***Address Correspondence to:** Scott Earley, Ph.D.
Department of Pharmacology/MS 0318
University of Nevada, Reno
School of Medicine, Manville 8
Reno, NV 89557-0318, USA
Phone: (775) 784-4117
Fax: (775) 784-1620
Email: searley@med.unr.edu

This PDF file includes:

- **Supplemental Figures: 1-12**
- **Supplemental Videos Legends: 1-3**

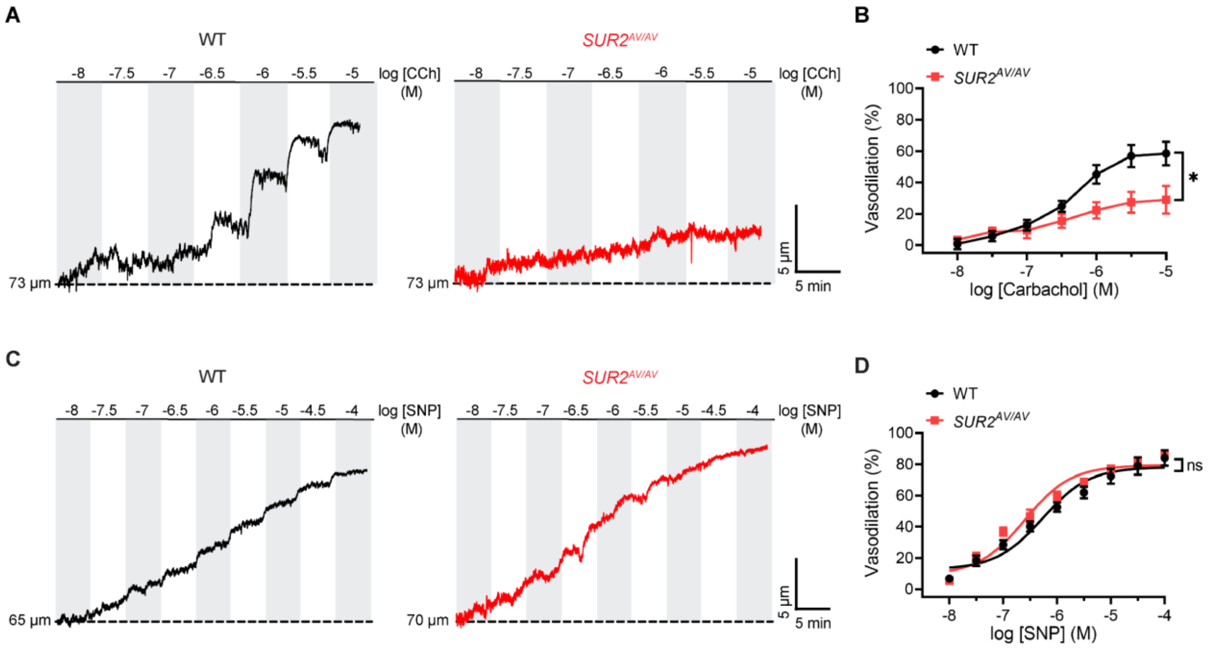

Supplemental Figure 1. Endothelium-dependent vasodilation is blunted in SUR2$^{AV/AV}$ mice. (**A**) Representative recordings and (**B**) summary data showing endothelium-dependent vasodilation induced by CCh (muscarinic receptor agonist) in mesenteric arteries from WT and SUR2$^{AV/AV}$ mice. Data are presented as means ± SEM (*n* = 8 vessels from four animals per group; **p* < 0.05, two-way ANOVA with Šídák's multiple comparisons test). (**C**) Representative recording and (**D**) summary data showing vasodilatory responses to the •NO donor SNP in isolated mesenteric arteries from WT and SUR2$^{AV/AV}$ mice. Data are presented as means ± SEM (*n* = 8 vessels from four animals per group; ns = not significant, two-way ANOVA with Šídák's multiple comparisons test).

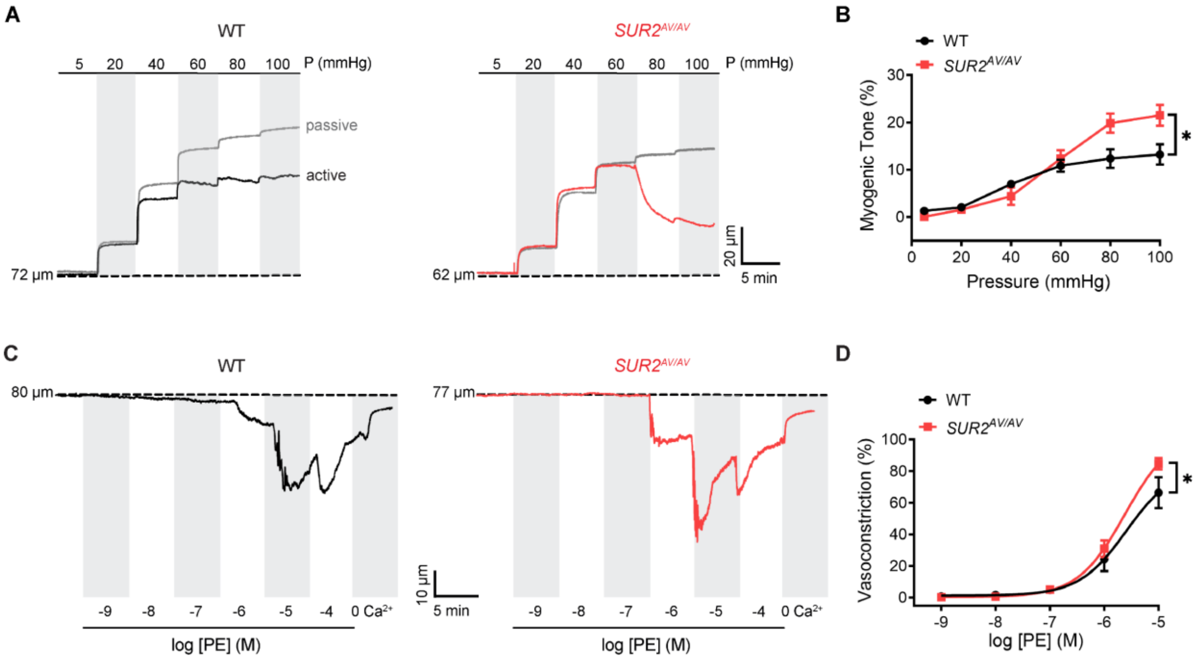

Supplemental Figure 2. Mesenteric arteries from SUR2$^{AV/AV}$ mice are hypercontractile. (**A**) Typical recording of the changes in lumen diameter of third-order mesenteric arteries from WT and SUR2$^{AV/AV}$ mice in response to increases in intraluminal pressure under active and passive (Ca^{2+}-free) conditions. (**B**) Summary data from WT and SUR2$^{AV/AV}$ mice. Data are presented as means ± SEM (n = 8 vessels from four mice per group; *p < 0.05, two-way ANOVA with Šídák's multiple comparisons test). (**C**) Representative recording and (**D**) summary data showing constriction of mesenteric arteries from WT and SUR2$^{AV/AV}$ mice in response to increasing concentrations of PE (α1-adrenoceptor agonist). Data are presented as means ± SEM (n = 6 vessels from four animals per group; *p < 0.05, two-way ANOVA with Šídák's multiple comparisons test).

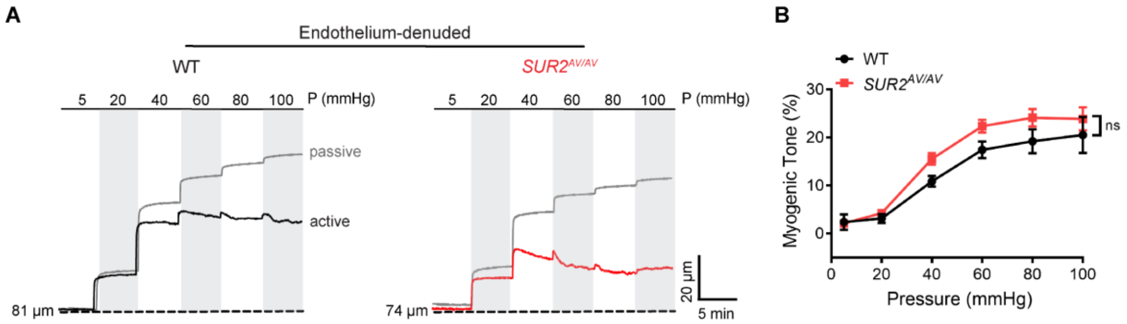

Supplemental Figure 3. Myogenic tone does not differ between WT and SUR2$^{AV/AV}$ mice in endothelium-denuded vessels. (A) Representative recording and (**B**) summary data of changes in lumen diameter in response to increases in intraluminal pressure under active and passive (Ca^{2+}-free) conditions in endothelium-denuded mesenteric arteries from WT and SUR2$^{AV/AV}$ mice. Data are presented as means ± SEM (*n* = 6 vessels from three animals per group; ns = not significant, two-way ANOVA with Šídák's multiple comparisons test).

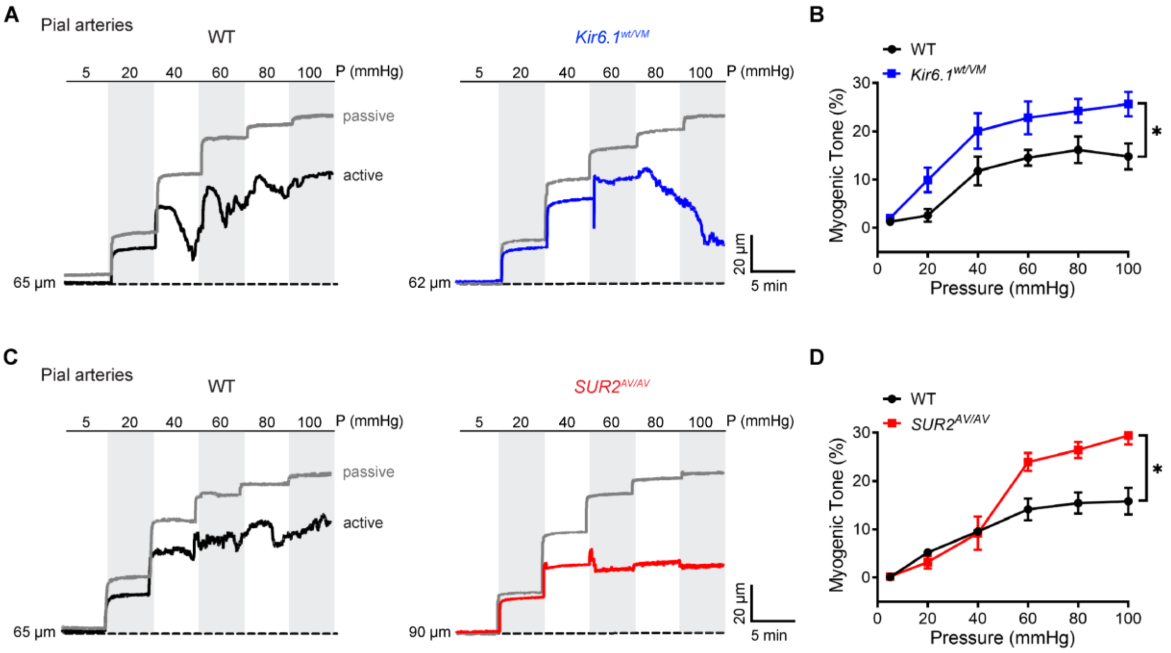

Supplemental Figure 4. Myogenic tone is elevated in cerebral pial arteries from Cantú mice. (A) Representative traces of the luminal diameter of isolated pial arteries from WT and Kir6.1$^{wt/VM}$ mice showing changes in lumen diameter in response to increases in intraluminal pressure under active and passive (Ca^{2+}-free) conditions. (**B**) Summary data of myogenic tone in pial arteries from WT and Kir6.1$^{wt/VM}$ mice. Data are presented as means ± SEM (n = 6 vessels from 4 animals per group; *$p < 0.05$, two-way ANOVA with Šídák's multiple comparisons test). (**C**) Representative traces of the luminal diameter of isolated pial arteries from WT and SUR$^{AV/AV}$ mice showing changes in lumen diameter in response to increases in intraluminal pressure under active and passive (Ca^{2+}-free) conditions. (**D**) Summary data of myogenic tone in pial arteries from WT and SUR$^{AV/AV}$ mice. Data are presented as means ± SEM (n = 6 vessels from 4 animals per group; *$p < 0.05$, two-way ANOVA with Šídák's multiple comparisons test).

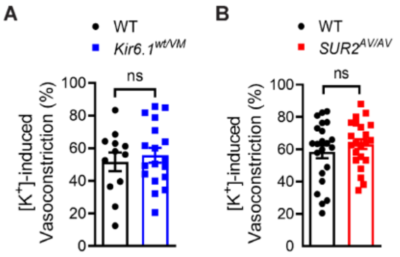

Supplemental Figure 5. Vasoconstriction in response to elevated extracellular [K^+] does not differ between WT and Cantú mice. Summary data showing constriction of isolated mesenteric arteries from Kir6.1$^{wt/VM}$ (**A**) and SUR2$^{AV/AV}$ (**B**) mice compared with WT controls in response to 60 mM KCl. Data are presented as means ± SEM (n = 12–22 arteries for WT and 18–22 arteries for Cantú mice from 10–12 animals; ns: not significant, Student’s *t-test*).

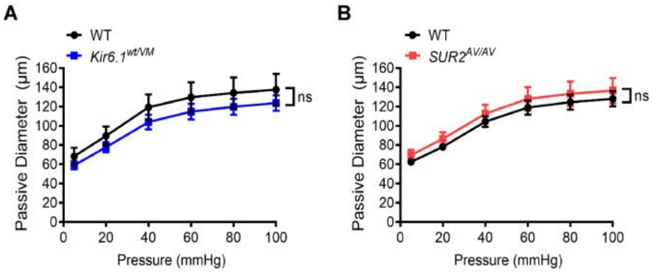

Supplemental Figure 6. The passive diameter of mesenteric arteries does not differ between WT and Cantú mice. Summary data showing the passive inner diameter of isolated mesenteric arteries from Kir6.1$^{wt/VM}$ (**A**) and SUR$^{AV/AV}$ (**B**) mice compared with WT controls under Ca^{2+}-free conditions (no added Ca^{2+}, 2 mM EGTA, 10 μM diltiazem). Data are presented as means ± SEM (n = 5–8 vessels from 4 animals per group; ns: not significant, two-way ANOVA with Šídák's multiple comparisons test).

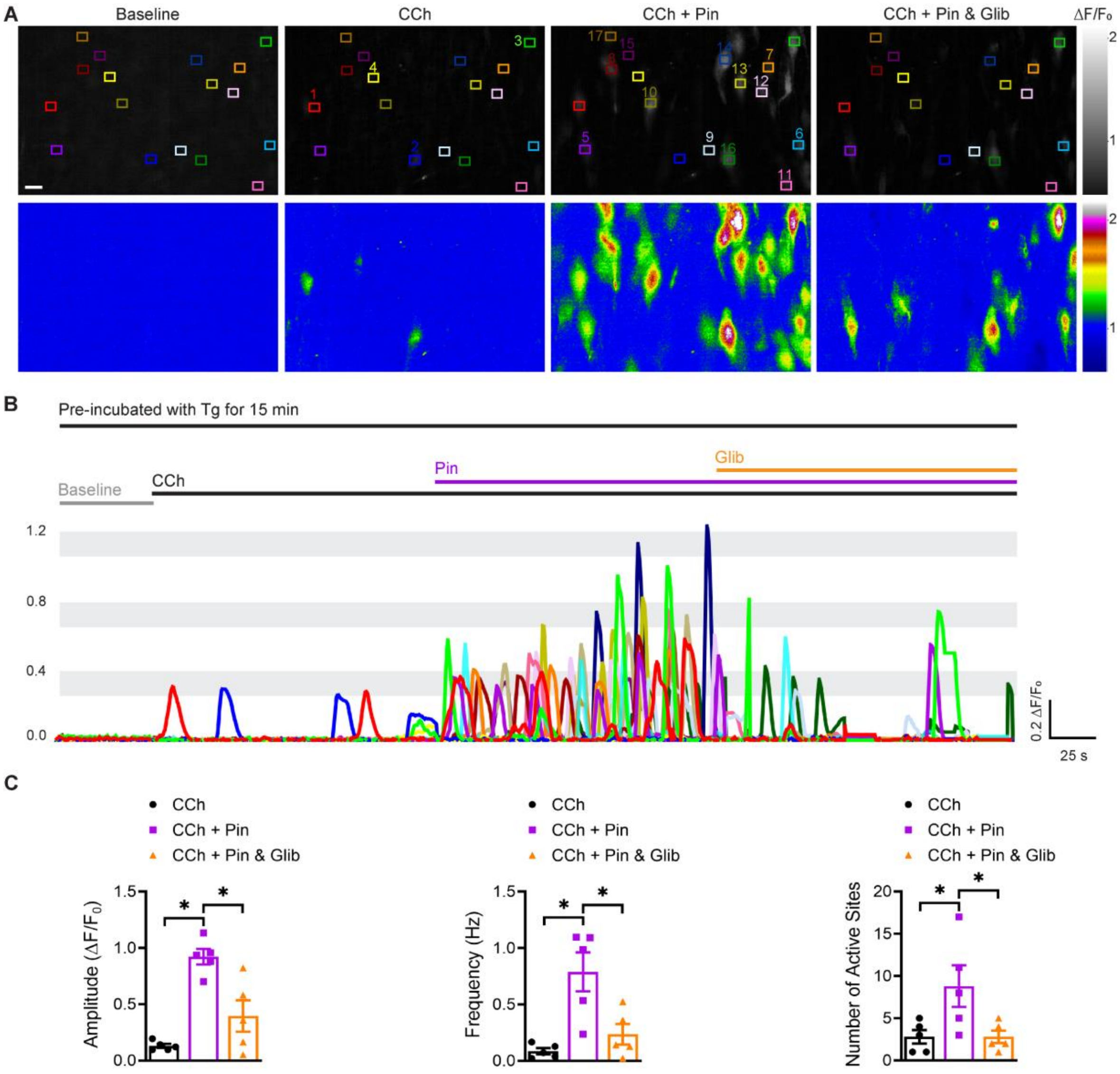

Supplemental Figure 7. KATP channel activation increases endothelial cell Ca^{2+} influx. (**A**) Representative grayscale and pseudocolored images of mesenteric arteries from Cdh5-GCaMP8 mice mounted *en face*. Arteries were pre-incubated with the sarcoendoplasmic reticulum Ca^{2+}-ATPase inhibitor thapsigargin (Tg; 2 µM) for 15 min. Recordings were performed initially under baseline conditions and then following initiation of Ca^{2+} signals by addition of CCh (10 µM). The tissue was then treated with pinacidil (Pin; 10 µM) followed by treatment with glibenclamide (Glib; 10 µM). Colored boxes show

regions of interest (ROIs) where Ca^{2+}-signaling events occurred. Scale bar = 10 µm. (**B**) Representative $\Delta F/F_0$ versus time plots of Ca^{2+} events from multiple Ca^{2+} event sites. (**C**) Summary data showing the effects of pinacidil and glibenclamide on the amplitude ($\Delta F/F_0$), frequency (Hz), and number of active sites. Data are presented as means ± SEM ($n = 5$ arteries from three animals; $*p < 0.05$, one-way ANOVA with Tukey's multiple comparisons test).

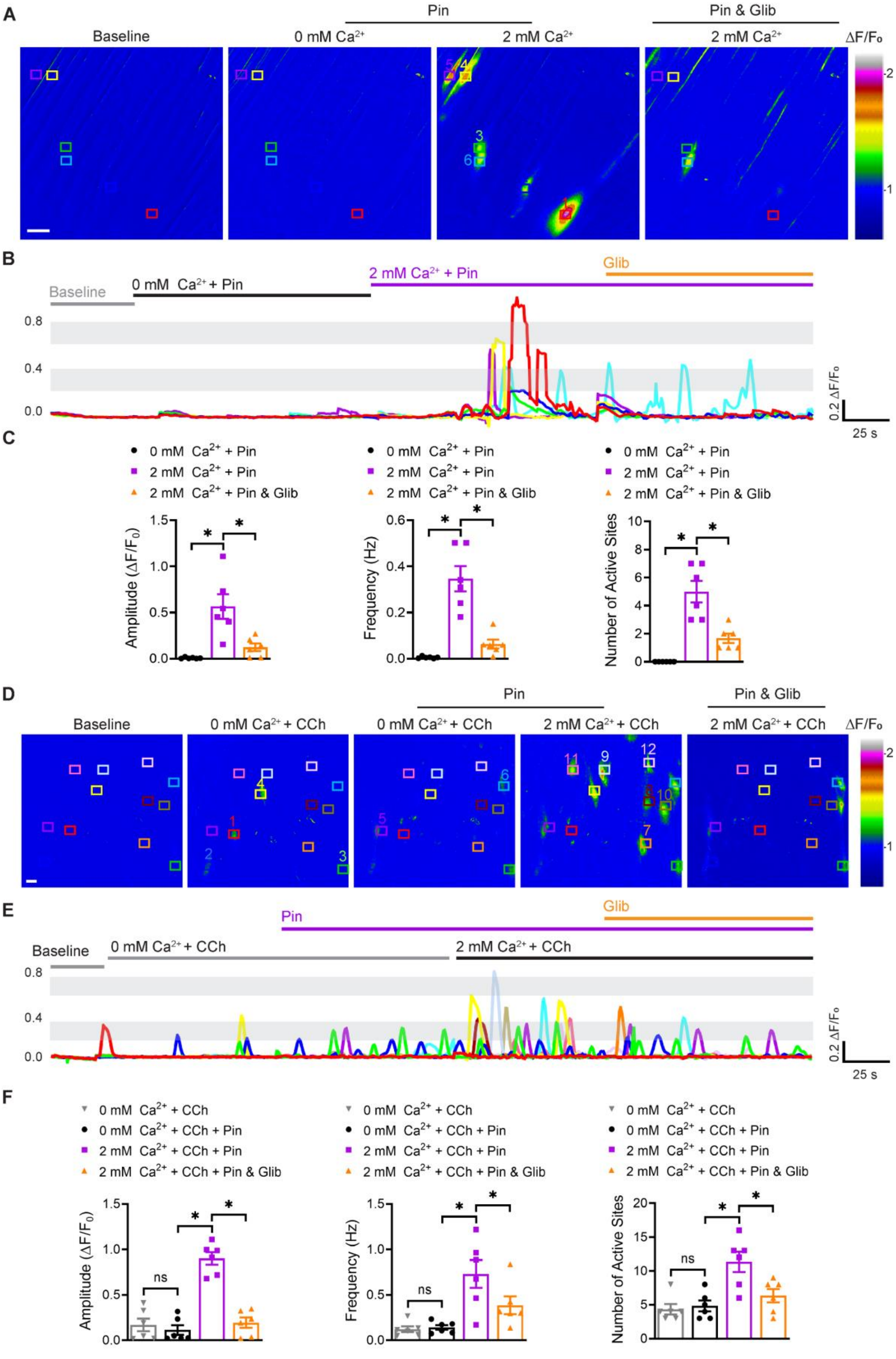
A
Baseline
Pin
0 mM Ca2+
2 mM Ca2+
Pin & Glib
2 mM Ca2+
ΔF/F0
B
Baseline
0 mM Ca2+ + Pin
2 mM Ca2+ + Pin
Glib
0.2 ΔF/F0
25 s
C
0 mM Ca2+ + Pin
2 mM Ca2+ + Pin
2 mM Ca2+ + Pin & Glib
Amplitude (ΔF/F0)
Frequency (Hz)
Number of Active Sites
D
Baseline
0 mM Ca2+ + CCh
Pin
0 mM Ca2+ + CCh
2 mM Ca2+ + CCh
Pin & Glib
2 mM Ca2+ + CCh
ΔF/F0
E
Pin
Glib
Baseline
0 mM Ca2+ + CCh
2 mM Ca2+ + CCh
0.2 ΔF/F0
25 s
F
0 mM Ca2+ + CCh
0 mM Ca2+ + CCh + Pin
2 mM Ca2+ + CCh + Pin
2 mM Ca2+ + CCh + Pin & Glib
Amplitude (ΔF/F0)
Frequency (Hz)
Number of Active Sites
ns

Supplemental Figure 8. Extracellular Ca^{2+} is required for pinacidil-induced Ca^{2+} influx. (**A**) Representative pseudocolored images of mesenteric arteries from *Cdh5*-GCaMP8 mice mounted *en face*. Endothelial cell Ca^{2+} signals were recorded under control conditions, then in the presence of pinacidil alone (Pin; 10 µM), with arteries bathed in a Ca^{2+}-free solution or a solution containing 2 mM extracellular Ca^{2+}, followed by treatment with the K_{ATP} channel blocker glibenclamide (Glib; 10 µM). Colored boxes show ROIs where Ca^{2+}-signaling events occurred. Scale bar = 10 µm. (**B**) Representative $\Delta F/F_0$ versus time plots of Ca^{2+} events from multiple Ca^{2+} event sites. (**C**) Summary data showing the effects of pinacidil on the amplitude ($\Delta F/F_0$), frequency (Hz), and number of active sites for Ca^{2+} signals in Ca^{2+}-free solution or a solution containing 2 mM extracellular Ca^{2+}. Data are presented as means ± SEM (n = 6 arteries from three animals; *$p < 0.05$, one-way ANOVA with Tukey's multiple comparisons test). (**D**) Representative pseudocolored images of mesenteric arteries from *Cdh5*-GCaMP8 mice mounted *en face.* (**E**) Representative $\Delta F/F_0$ versus time plots of Ca^{2+} events at multiple sites. For CCh-induced Ca^{2+} signaling, events were recorded without or with pinacidil (Pin; 10 µM) in the absence of extracellular Ca^{2+} or in a solution containing 2 mM extracellular Ca^{2+}, followed by treatment with glibenclamide (Glib; 10 µM). Colored boxes indicate ROIs with active Ca^{2+} signals. Scale bar = 10 µm. (**F**) Summary data showing the effects of pinacidil at 0 mM or 2 mM extracellular Ca^{2+} in the presence of CCh. The amplitude ($\Delta F/F_0$), frequency (Hz), and number of active sites for Ca^{2+} signals were analyzed. Data are presented as means ± SEM (n = 6 arteries from 3 mice; *$p < 0.05$, two-way ANOVA with Tukey's multiple comparisons test).

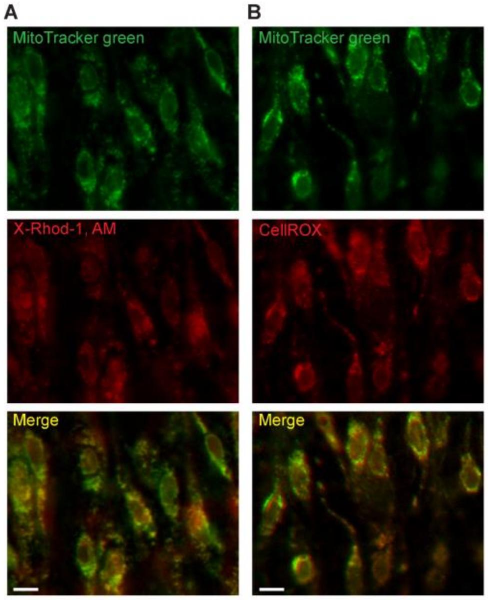

Supplemental Figure 9. **Specificity of mitochondrial labeling.** Representative confocal images of mesenteric arteries from WT mice mounted *en face*, co-labeled with MitoTracker green to identify mitochondria and (**A**) X-Rhod-1 or (**B**) CellRox. Scale bar = 10 µm.

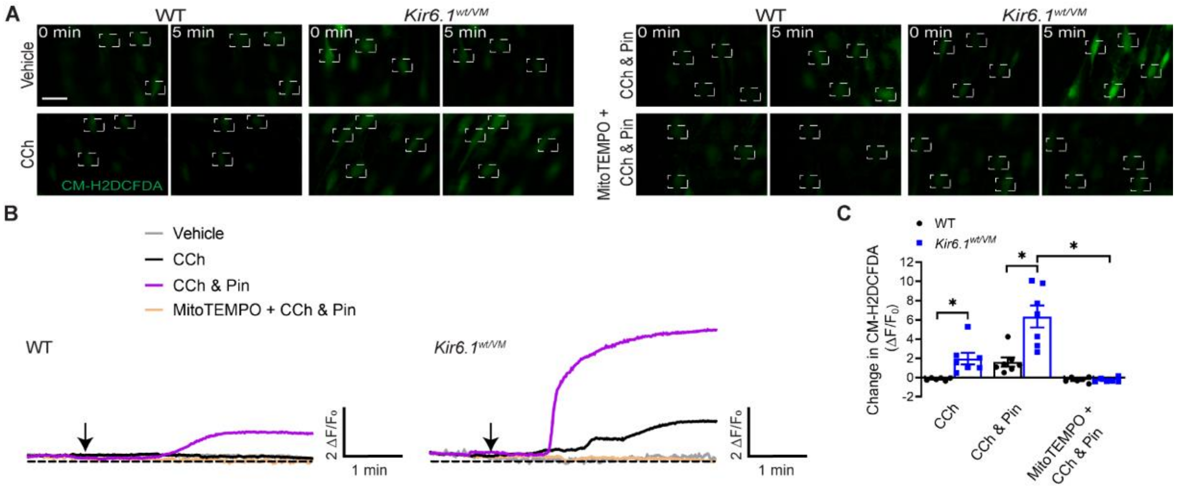

Supplemental Figure 10. Cytosolic ROS generation is increased in the endothelium of Cantú mice. (**A**) Representative images of mesenteric arteries from WT and Kir6.1$^{wt/VM}$ mice mounted *en face* and loaded with the general oxidative stress indicator CM-H2DCFDA. Changes in CM-H2DCFDA fluorescence were imaged following treatment with CCh (10 μM), pinacidil (Pin; 10 μM), and the mitochondrial ROS scavenger mitoTEMPO (5 μM). Boxes show regions of interest (ROIs). Scale bar = 10 μm. (**B**) Representative $\Delta F/F0$ versus time plots of the change in fluorescence intensity under each condition. Arrows indicate application of the agonist. (**C**) Summary data showing the changes in CM-H2DCFDA fluorescence intensity. Data are presented as means ± SEM (*n* = 6 to 7 arteries from 3 animals per group; **p* < 0.05, two-way ANOVA with Tukey's multiple comparisons test).

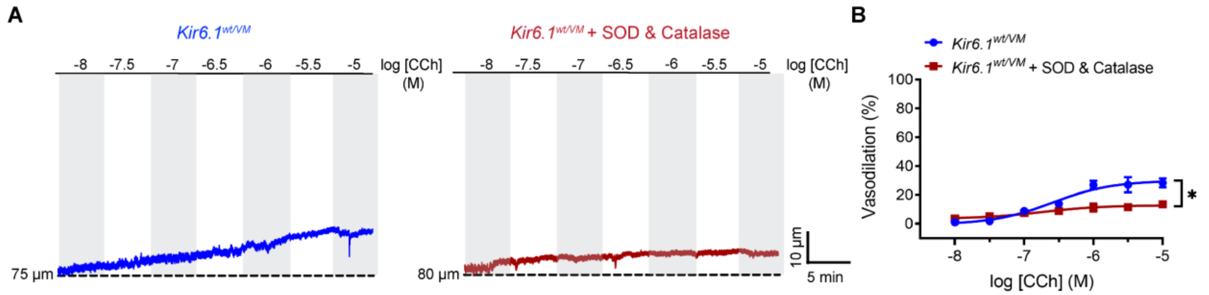

Supplemental Figure 11. Extracellular ROS inhibition does not restore endothelium-dependent dilation in mesenteric arteries from Kir6.1$^{wt/VM}$ mice. (**A**) Representative recordings and (**B**) summary data showing endothelium-dependent dilation evoked by CCh in mesenteric arteries from Kir6.1$^{wt/VM}$ mice before and after treatment with the extracellular ROS scavengers SOD (500 U/ml) and catalase (500 U/ml). Data are presented as means ± SEM (n = 5 vessels from 4 animals per group; *p < 0.05, two-way ANOVA with Šídák's multiple comparisons test).

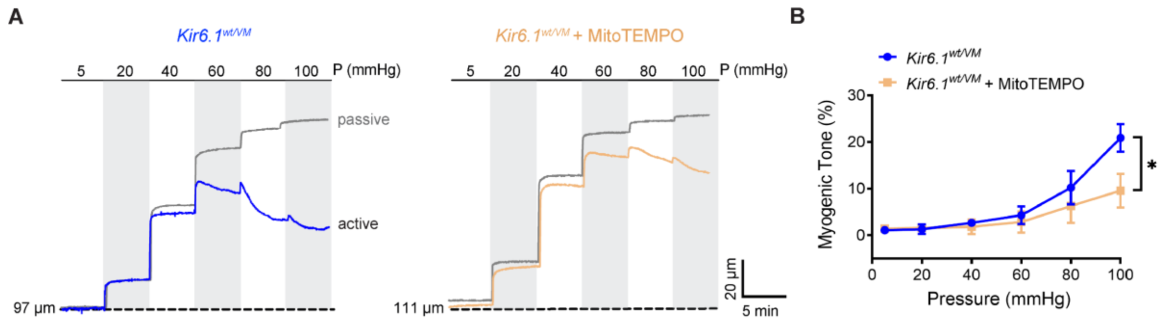

Supplemental Figure 12. Blocking mitochondrial ROS generation reduces the hypercontractility of arteries from Kir6.1$^{wt/VM}$ mice. (**A**) Representative recording showing the change in lumen diameter of mesenteric arteries from Kir6.1$^{wt/VM}$ mice in response to increases in intraluminal pressure under active and passive (Ca^{2+}-free)

conditions. The trace on the right shows a representative recording of an artery treated with mitoTEMPO. (**B**) Summary data showing myogenic tone in mesenteric arteries from Kir6.1$^{wt/VM}$ mice treated with mitoTEMPO or vehicle. Data are presented as means ± SEM (*n* = 6-7 vessels from 5 animals per group; **p* < 0.05, two-way ANOVA with Šídák's multiple comparisons test).

Supplemental Videos Legends

Supplemental Video 1. Pharmacological activation of K_{ATP} channels increases CCh-induced Ca^{2+}-signaling events in mesenteric arteries from *Cdh5*-GCaMP8 mice. Ca^{2+} signals were recorded in the presence of CCh (10 µM), and following treatment with pinacidil (Pin; 10 µM) and glibenclamide (Glib; 10 µM).

Supplemental Video 2. Extracellular Ca^{2+} is required for K_{ATP} channel-induced increases in Ca^{2+} influx. Ca^{2+} events were recorded in the endothelium of mesenteric arteries from *Cdh5*-GCaMP8 in the presence of pinacidil (Pin; 10 µM) in a Ca^{2+}-free solution or a solution containing 2 mM extracellular Ca^{2+}.

Supplemental Video 3. CCh-induced Ca^{2+}-signaling activity is increased in mesenteric arteries from Kir6.1$^{wt/VM}$ mice. Endothelial Ca^{2+} signals were recorded from a mesenteric artery isolated from a *Cdh5*-GCaMP8 x Kir6.1$^{wt/VM}$ mouse in the presence of CCh (10 µM) and following treatment with pinacidil (Pin; 10 µM) and glibenclamide (Glib; 10 µM).
